# Supplementary material for: Dilation and Evacuation Simulation Model for Learners and Providers Who Offer Abortion Care
Source: MedEdPORTAL. 2025 May 9;21:11525. doi: 10.15766/mep_2374-8265.11525 (PMC12062342; doi:10.15766/mep_2374-8265.11525)
Supplement: Supplementary file 1 — Simulation Materials and Assembly.docxPresimulation Survey.docxIntroductory Lecture.pptxD&E Simulation Demonstration Video.mp4Postsimulation Survey.docx [file mep_2374-8265.11525-s001.zip › A. Simulation Materials and Assembly.docx]

**Materials and Assembly Required for Dilation and Extraction (D&E) Simulation Model**

(The simulation models should be assembled prior to the start of the introductory lecture and simulation)

Material Required (for one D&E simulation model):

- - Plastic juice carton (1 gallon), with neck opening measuring approximately 1.5 inches (3.8 centimeters) in diameter
  - Utility knife
  - Tape measure
  - Carpet foam (or similar spongy material)
  - Scissors
  - Double-sided tape
  - Duct tape
  - Cornish hen (raw)
  - Sopher forceps
  - Gloves (recommended)
  - Absorbent pad or plastic sheet (recommended)
  - Flat surface

Assembly:


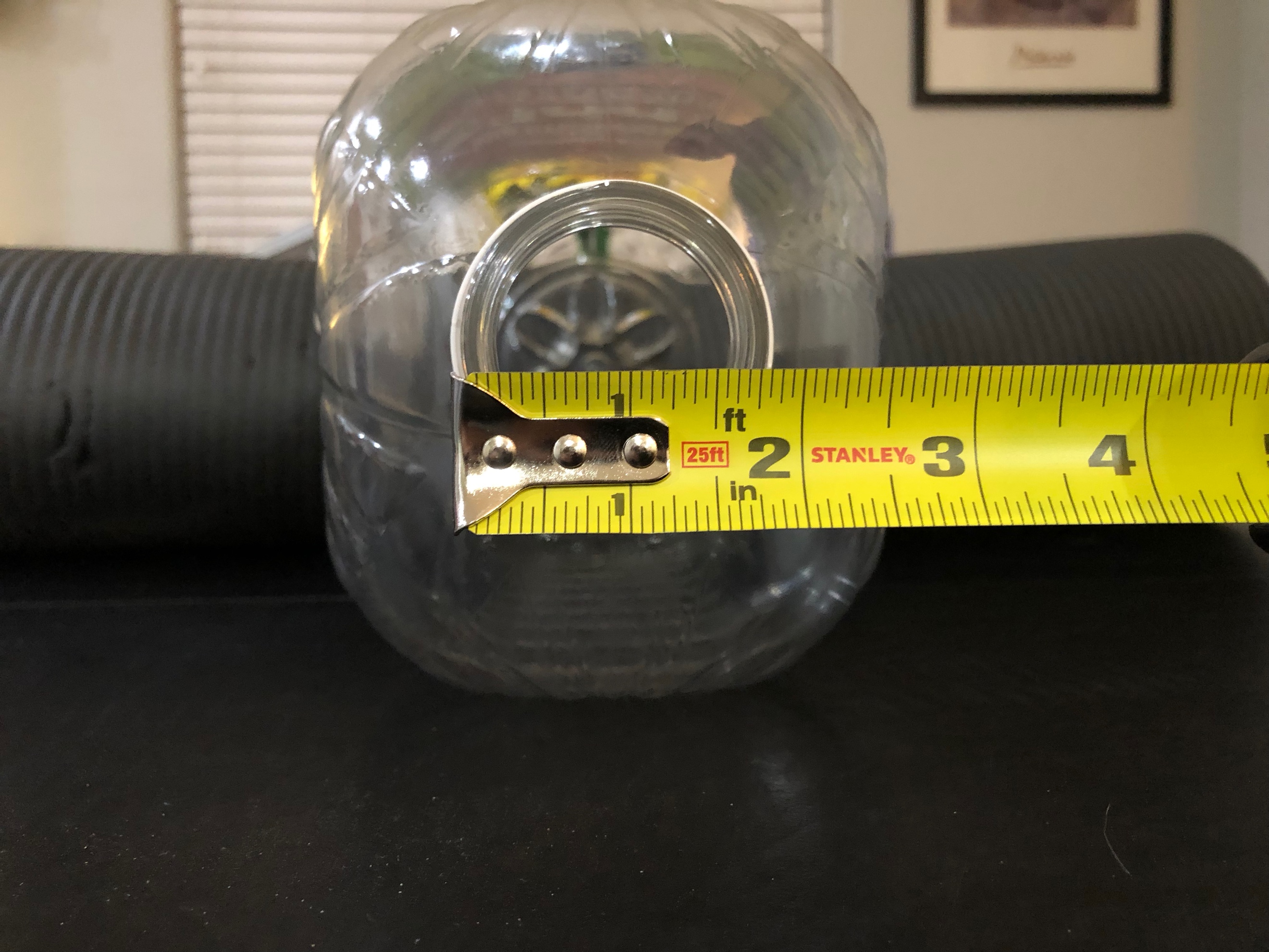


1. Thoroughly wash and dry a plastic juice carton (1 gallon).
2. The neck of the container should measure approximately 1.5 inches (3.8 centimeters). This simulates a dilated cervix.


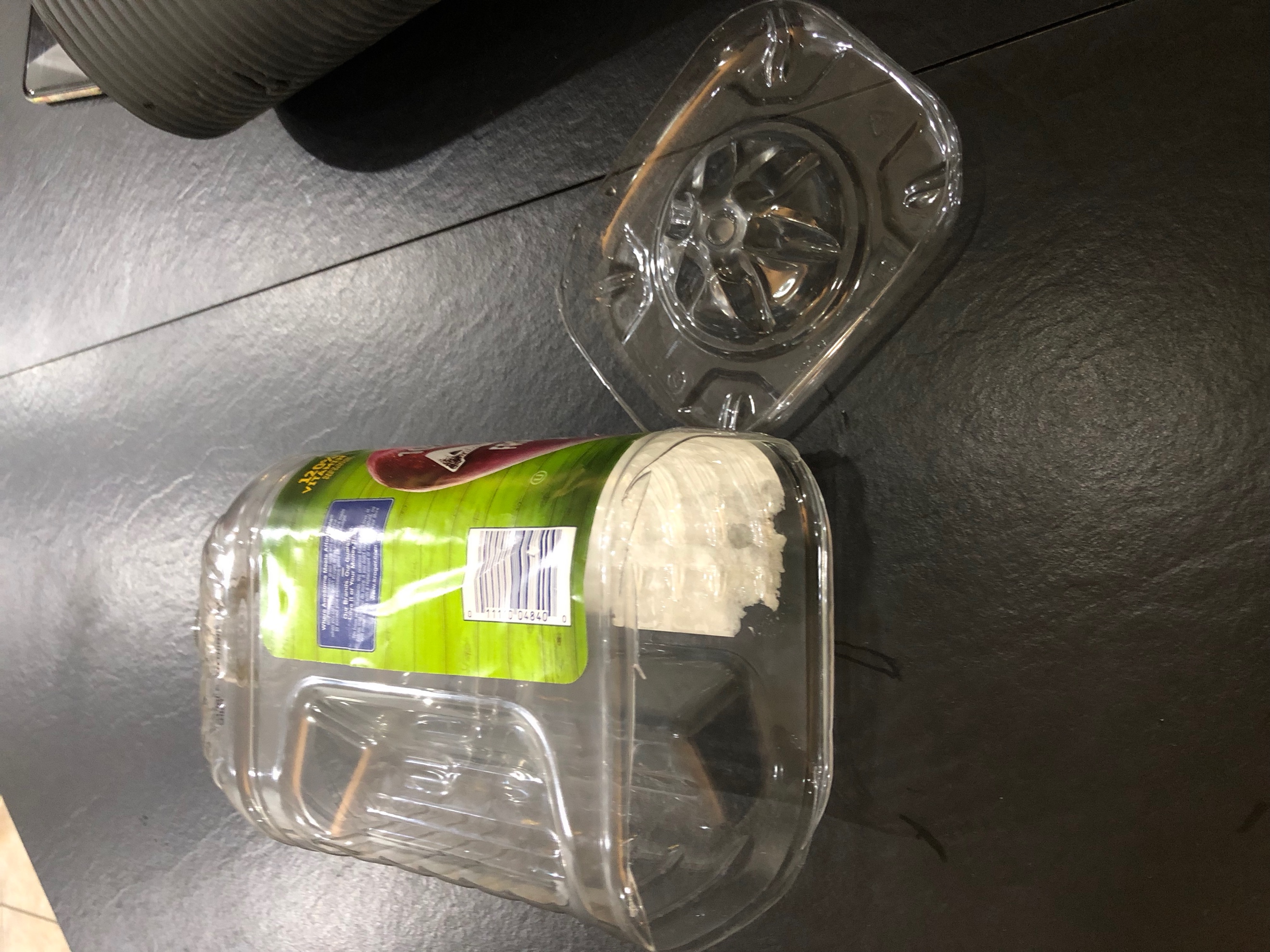

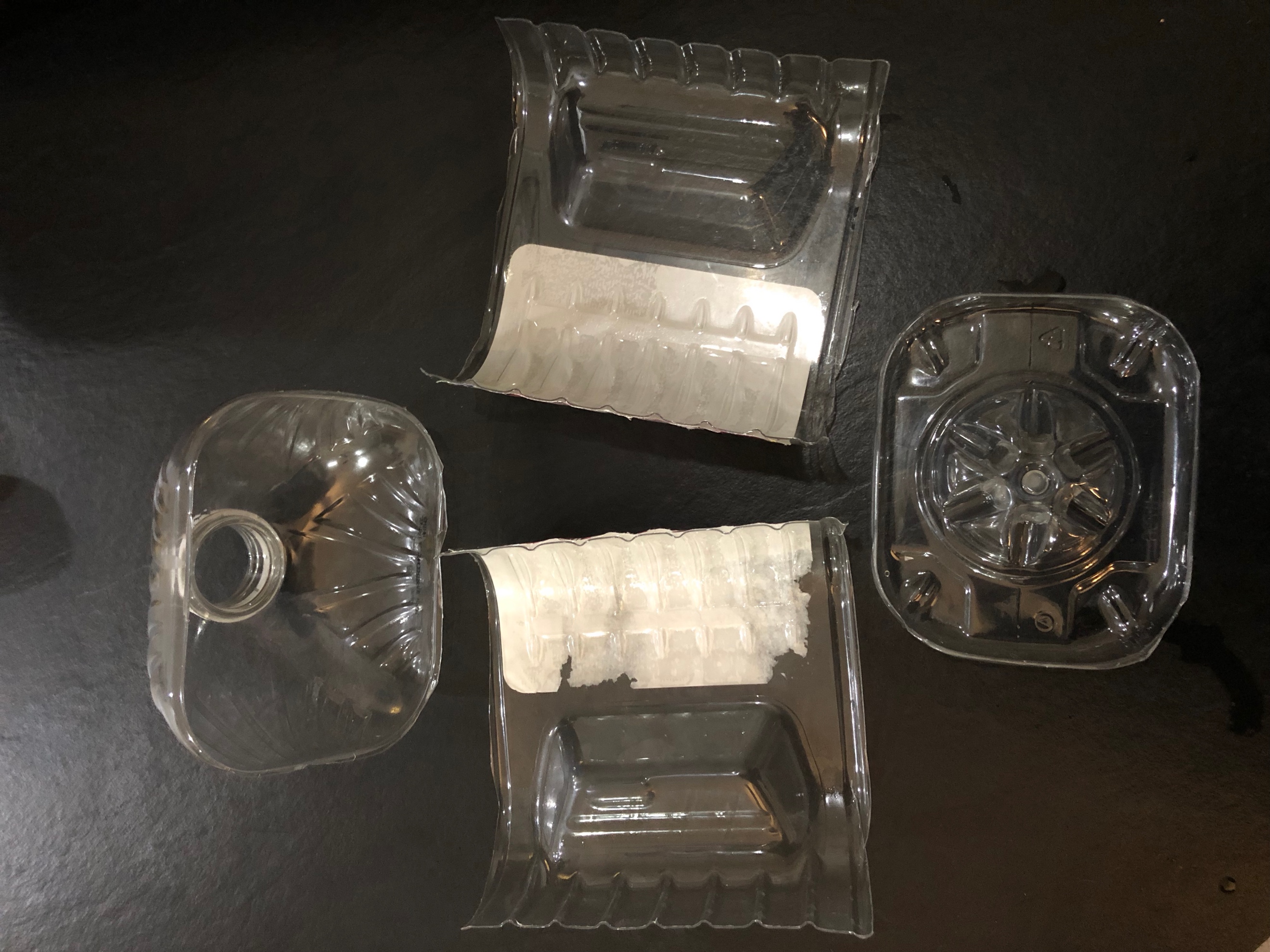


1. Using a utility knife, completely remove the bottom of the carton.
2. Completely remove the neck of the carton.
3. Cut the remaining middle section in half as demonstrated above.


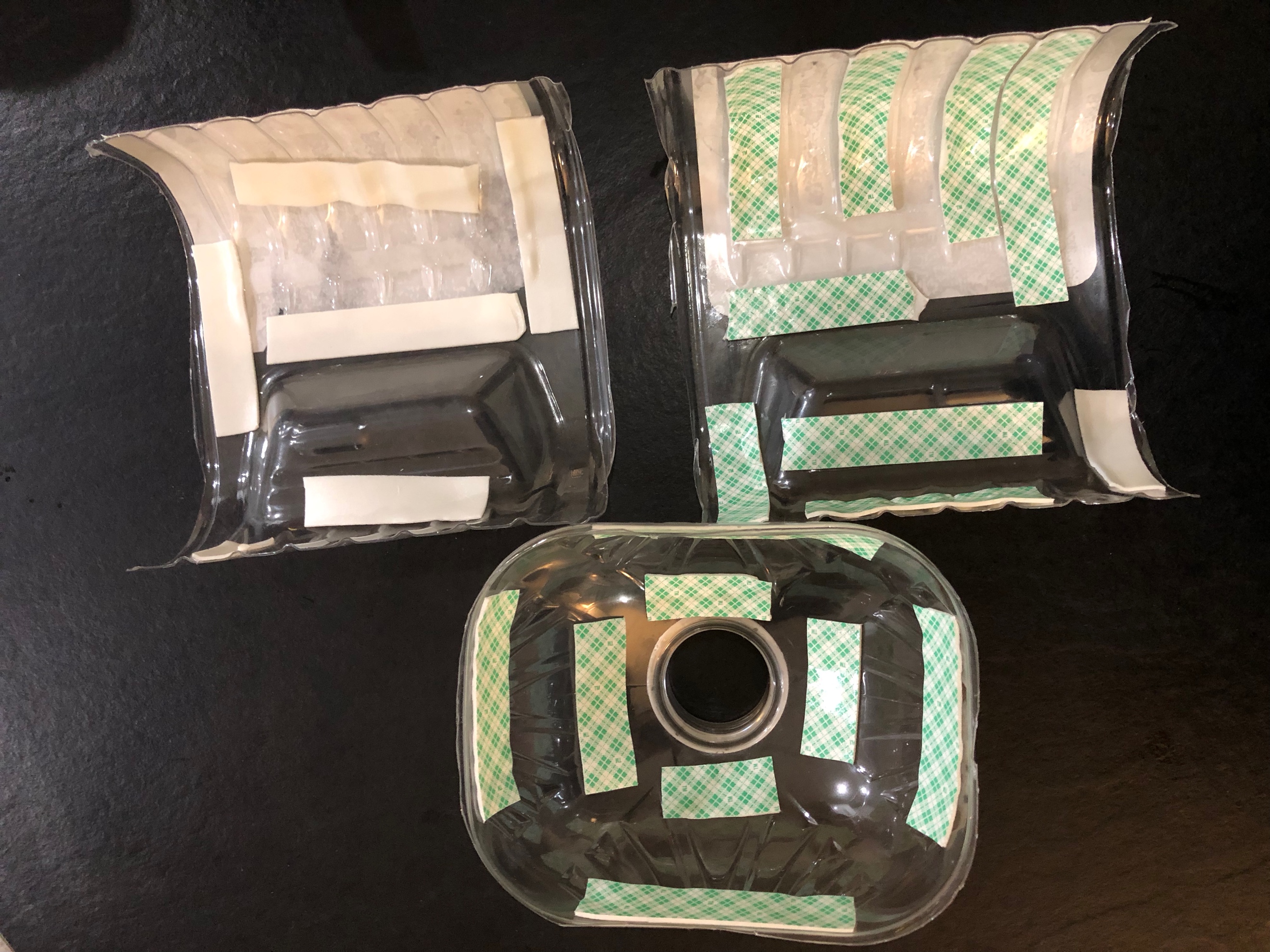


1. Cover the inner surfaces of all four carton pieces with double-sided tape.


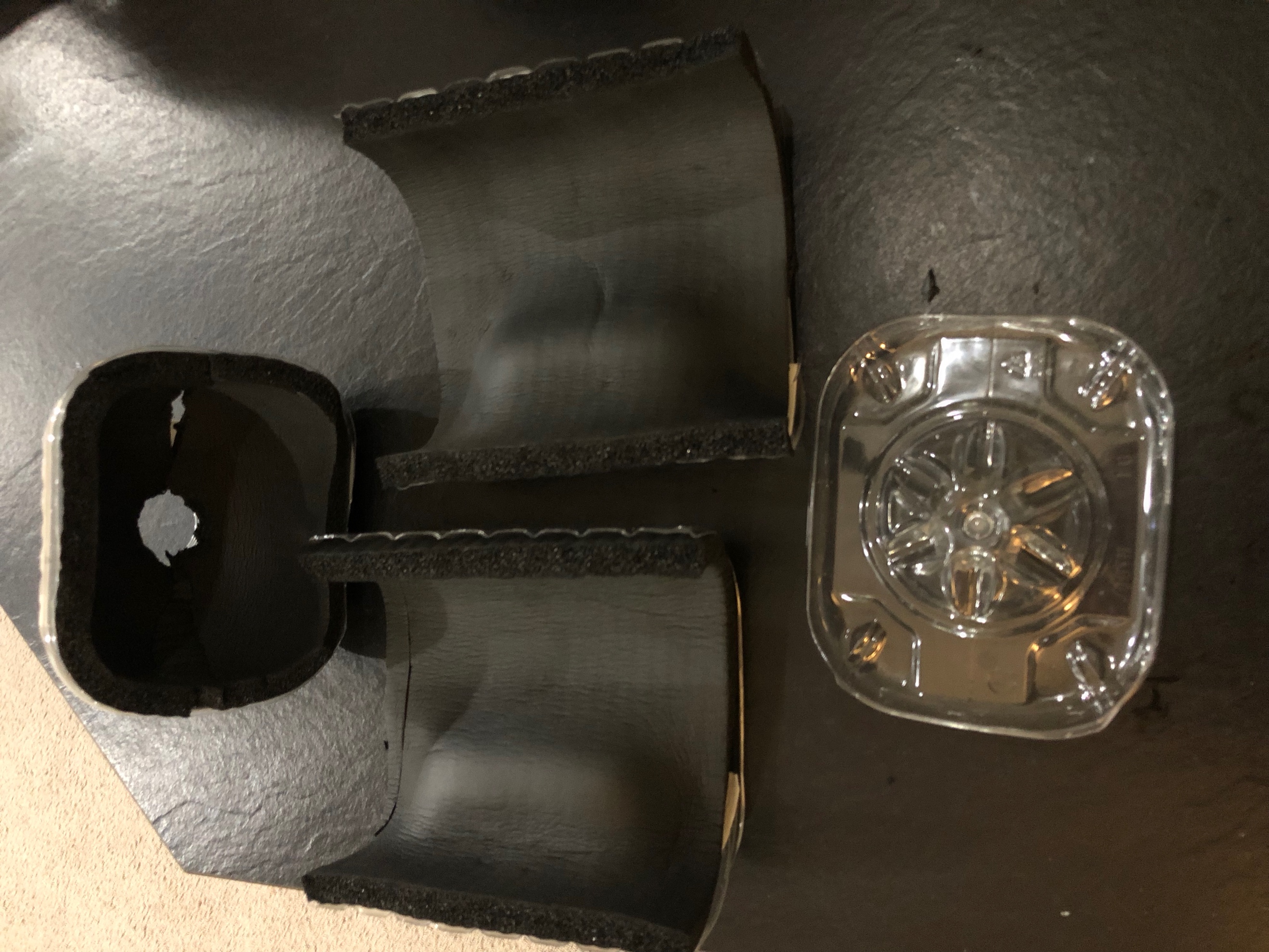


1. Measure and cut the carpet foam to fit the inner surface of each carton piece, and adhere the foam to the double-sided tape. All four pieces of the carton should be covered in foam. This simulates endometrium.


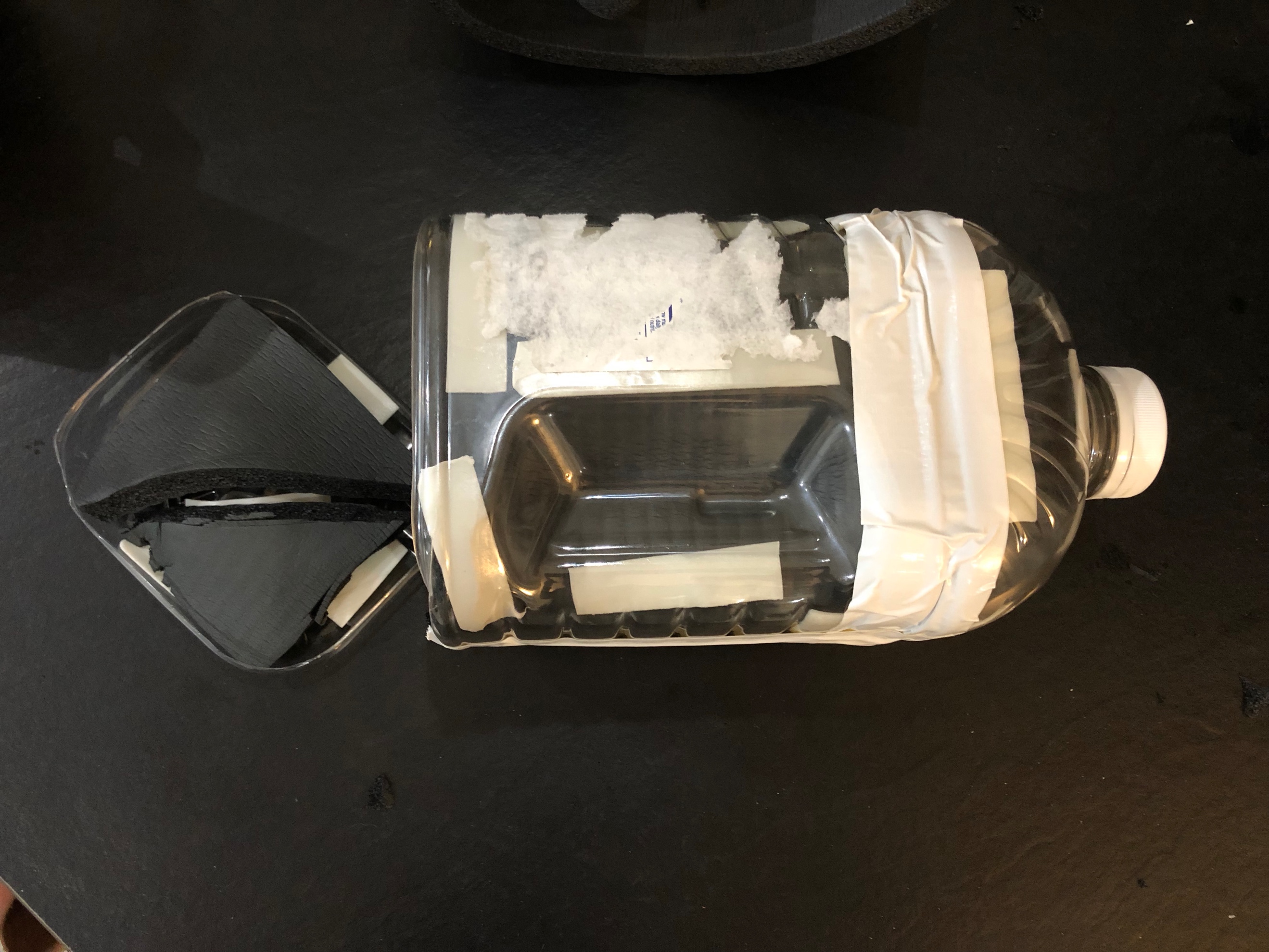


1. Using duct tape, reassemble the neck and middle sections of the carton. These should not be able to come apart again.


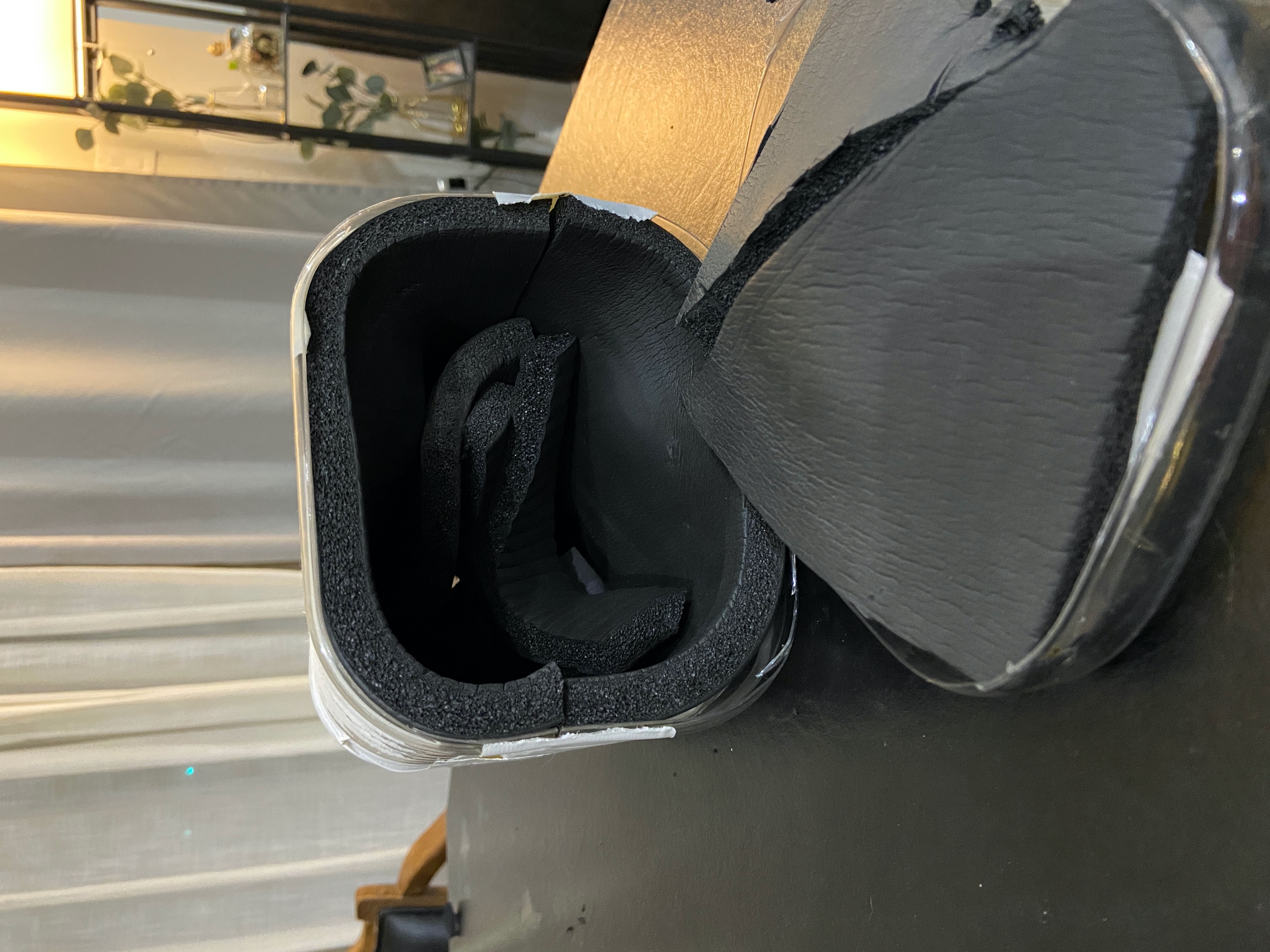


1. Attach the bottom piece to the rest of the carton on one side only, forming a hinge so it can be opened and closed.
   1. Optional: Place additional foam inside the carton to simulate placental tissue.


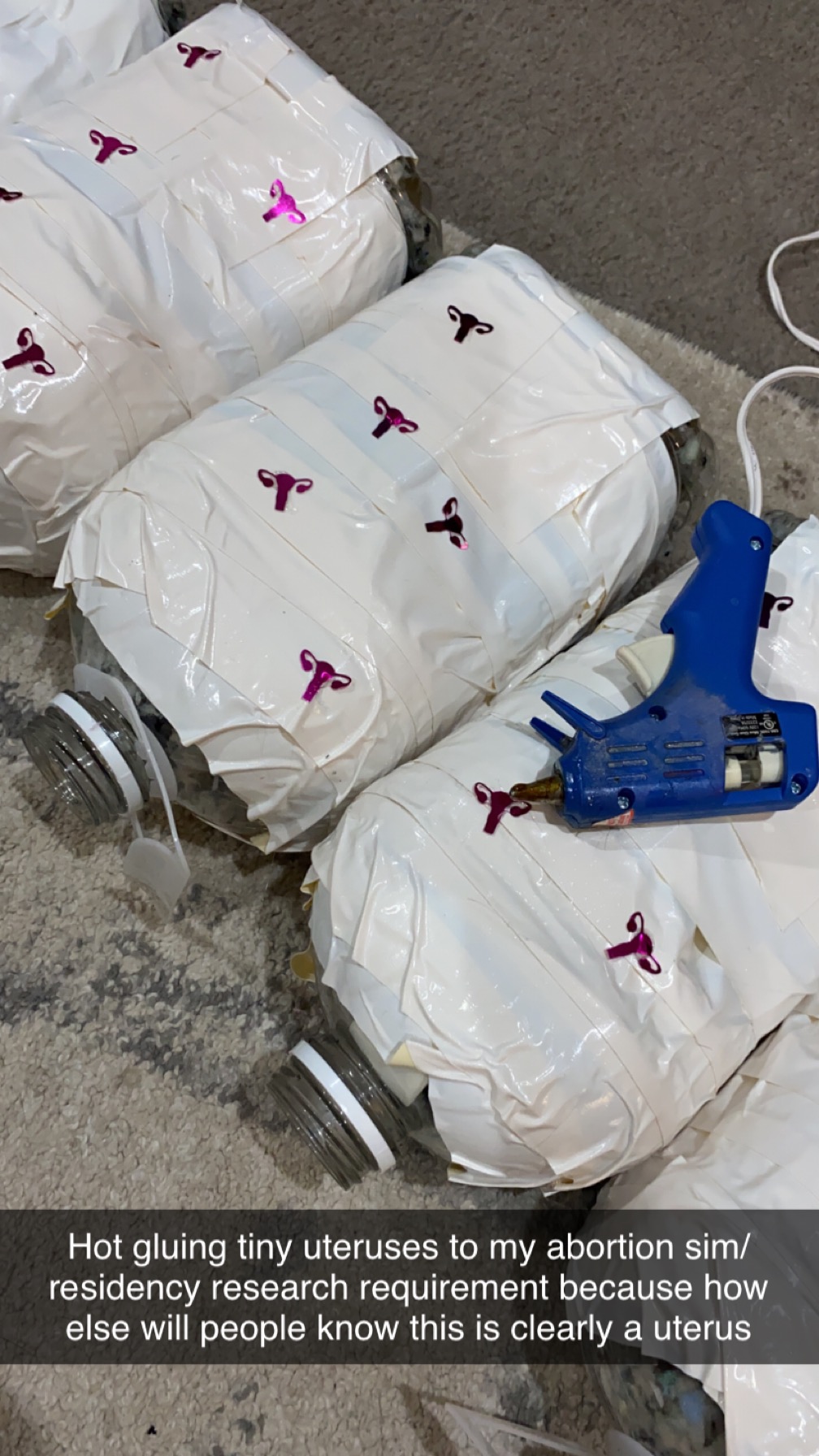


1. Completely cover the tops and sides of the carton with duct tape to obscure the contents inside (foam and Cornish hen).
2. Place the Cornish hen inside, through the bottom of the carton which can be opened and closed. This simulates the fetus. The hen should not be disarticulated prior to placing it inside the container.


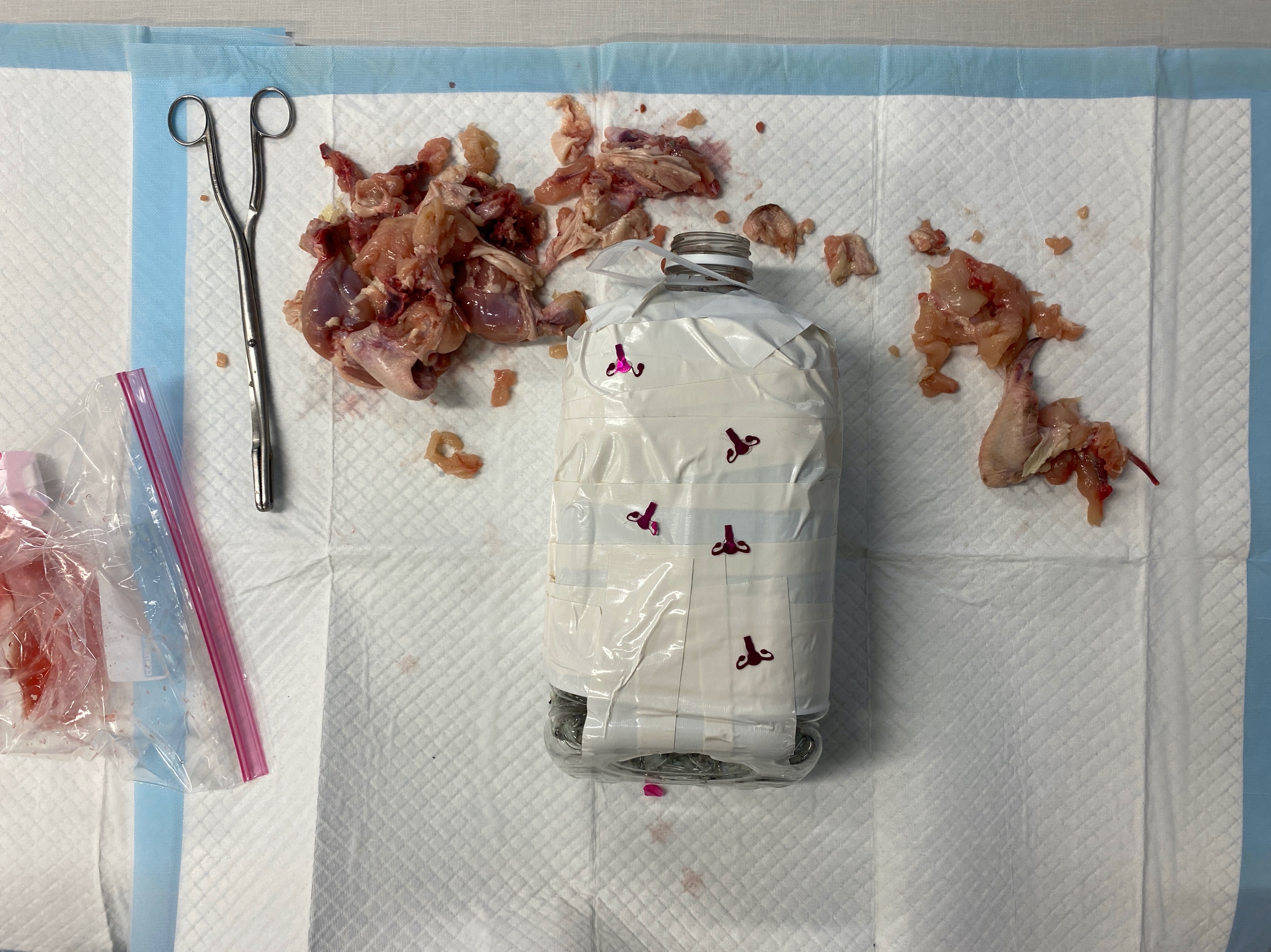


1. Set up the D&E model on a flat surface with an absorbent pad or plastic sheet underneath.
2. The person performing the D&E should stand in front of the neck of the carton and use Sopher forceps to methodically remove the hen (and loose foam).
3. Another person should stand at the base of the carton to firmly hold it in place against the table.
4. Once the hen (and loose foam) has been removed in its entirety, the inside of the carton should be examined to evaluate for any pieces of adhered carpet foam that have been removed. This would simulate injured endometrial tissue.
5. Each model can be reused by replacing a fresh Cornish hen between participants. If desired, the models can be reused across multiple instances if each carton is disassembled, the carpet foam is removed and discarded, the carton is thoroughly washed and dried, and the carpet foam is replaced prior to reassembly.

All images are author owned
