# Supplementary material for: Dilation and Evacuation Simulation Model for Learners and Providers Who Offer Abortion Care
Source: MedEdPORTAL. 2025 May 9;21:11525. doi: 10.15766/mep_2374-8265.11525 (PMC12062342; doi:10.15766/mep_2374-8265.11525)
Supplement: Supplementary file 1 — Simulation Materials and Assembly.docxPresimulation Survey.docxIntroductory Lecture.pptxD&E Simulation Demonstration Video.mp4Postsimulation Survey.docx [file mep_2374-8265.11525-s001.zip › B. Presimulation Survey.docx]

D&E Pre-Simulation Survey

(To be administered to participants prior to the introductory lecture and simulation)

1. What level of training are you currently in?

- MS3
- MS4
- PGY1
- PGY2
- PGY3
- PGY4
- Other Resident

2. Before today, have you ever participated in a Dilation and Evacuation simulation?

- Yes
- No

3. Have you ever performed all or part of a Dilation and Evacuation in an outpatient or inpatient setting?

- Yes
- No

4. How comfortable do you feel in your abilities to perform a Dilation and Evacuation?

- Extremely uncomfortable
- Somewhat uncomfortable
- Neither comfortable nor uncomfortable
- Somewhat comfortable
- Extremely comfortable

5. How comfortable do you feel in your abilities to recognize post-abortion complications?

- Extremely uncomfortable
- Somewhat uncomfortable
- Neither comfortable nor uncomfortable
- Somewhat comfortable
- Extremely comfortable

6. If not legally prohibited, do you intend to provide abortion care in your future practice?

- Yes
- No
- Unsure
- Not applicable (medical student or resident not interested in OBGYN)

7. If yes, do you intend to perform Dilation and Evacuation?

- Yes
- No
- Unsure
- Not applicable (medical student or resident not interested in OBGYN)

8. How likely would you be to perform Dilation and Evacuation procedures in future practice if you were to receive more clinical training during residency?

- I would not provide D&Es even if exposed to more clinical experiences
- Somewhat more likely
- Much more likely
- I would definitely provide D&Es if exposed to more clinical experiences
- Unsure
- Not applicable (medical student or resident not interested in OBGYN)

9. How likely would you be to perform Dilation and Evacuation procedures in future practice if you were to receive more simulated training during residency?

- I would not provide D&Es even if exposed to more simulated experiences
- Somewhat more likely
- Much more likely
- I would definitely provide D&Es if exposed to more simulated experiences
- Unsure
- Not applicable (medical student or resident not interested in OBGYN)
