# Supplementary material for: Dilation and Evacuation Simulation Model for Learners and Providers Who Offer Abortion Care
Source: MedEdPORTAL. 2025 May 9;21:11525. doi: 10.15766/mep_2374-8265.11525 (PMC12062342; doi:10.15766/mep_2374-8265.11525)
Supplement: Supplementary file 1 — Simulation Materials and Assembly.docxPresimulation Survey.docxIntroductory Lecture.pptxD&E Simulation Demonstration Video.mp4Postsimulation Survey.docx [file mep_2374-8265.11525-s001.zip › C. Introductory Lecture.pptx]

## Slide 1
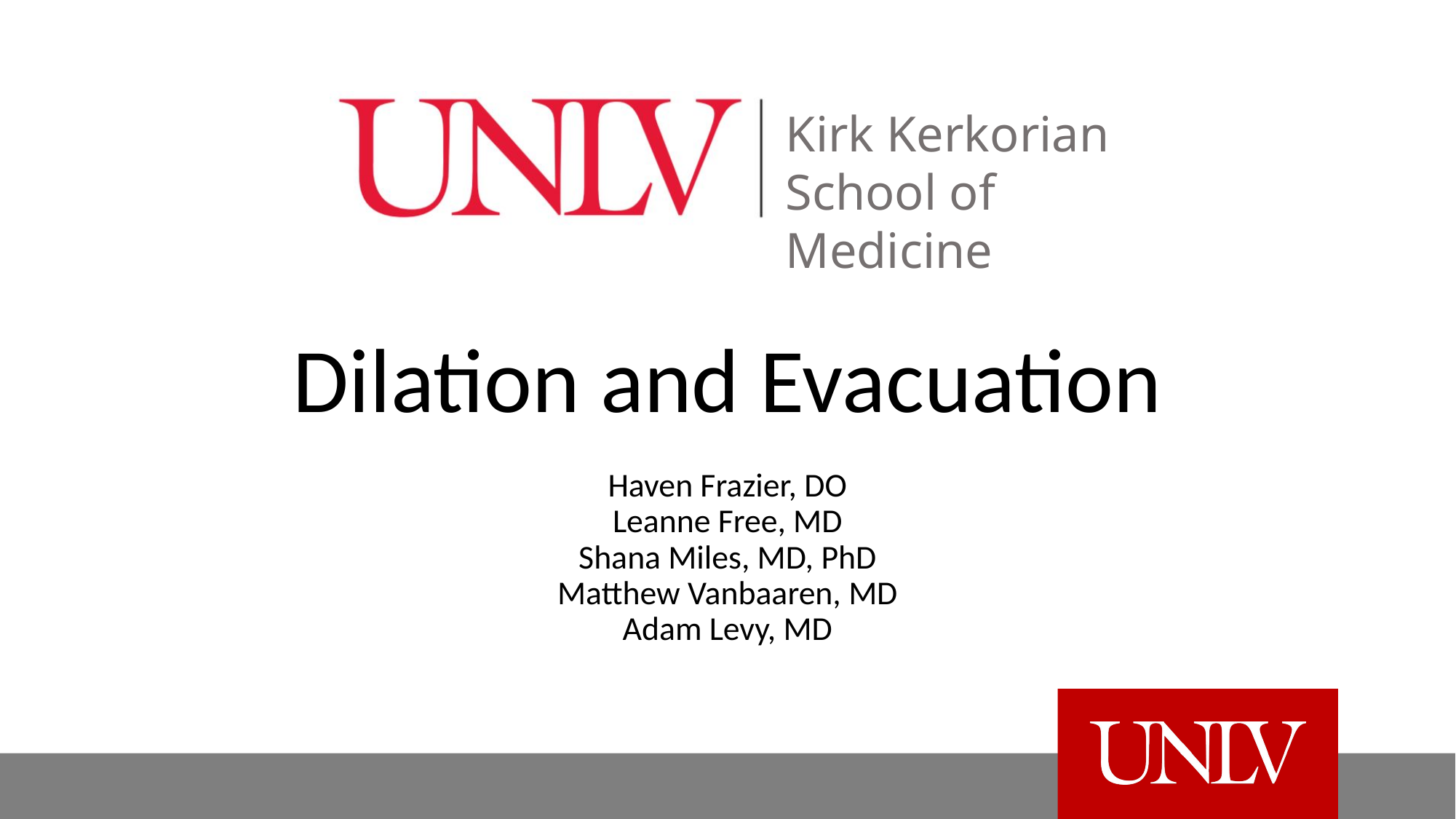

Kirk Kerkorian
School of Medicine
# Dilation and Evacuation Haven Frazier, DO Leanne Free, MD Shana Miles, MD, PhD Matthew Vanbaaren, MD Adam Levy, MD

## Slide 2
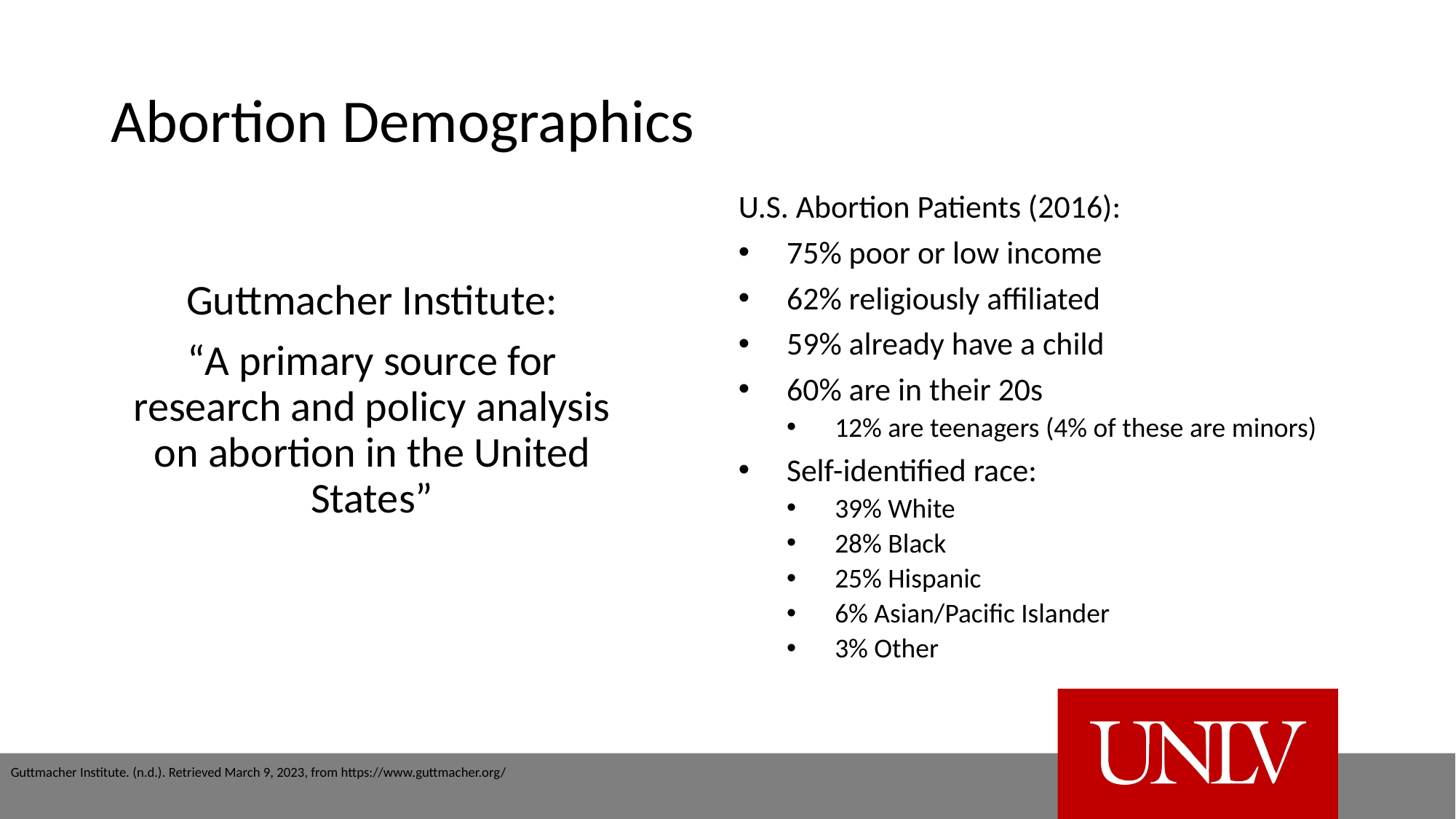

# Abortion Demographics
U.S. Abortion Patients (2016):
75% poor or low income
62% religiously affiliated
59% already have a child
60% are in their 20s
12% are teenagers (4% of these are minors)
Self-identified race:
39% White
28% Black
25% Hispanic
6% Asian/Pacific Islander
3% Other
Guttmacher Institute:
“A primary source for research and policy analysis on abortion in the United States”
Guttmacher Institute. (n.d.). Retrieved March 9, 2023, from https://www.guttmacher.org/

## Slide 3
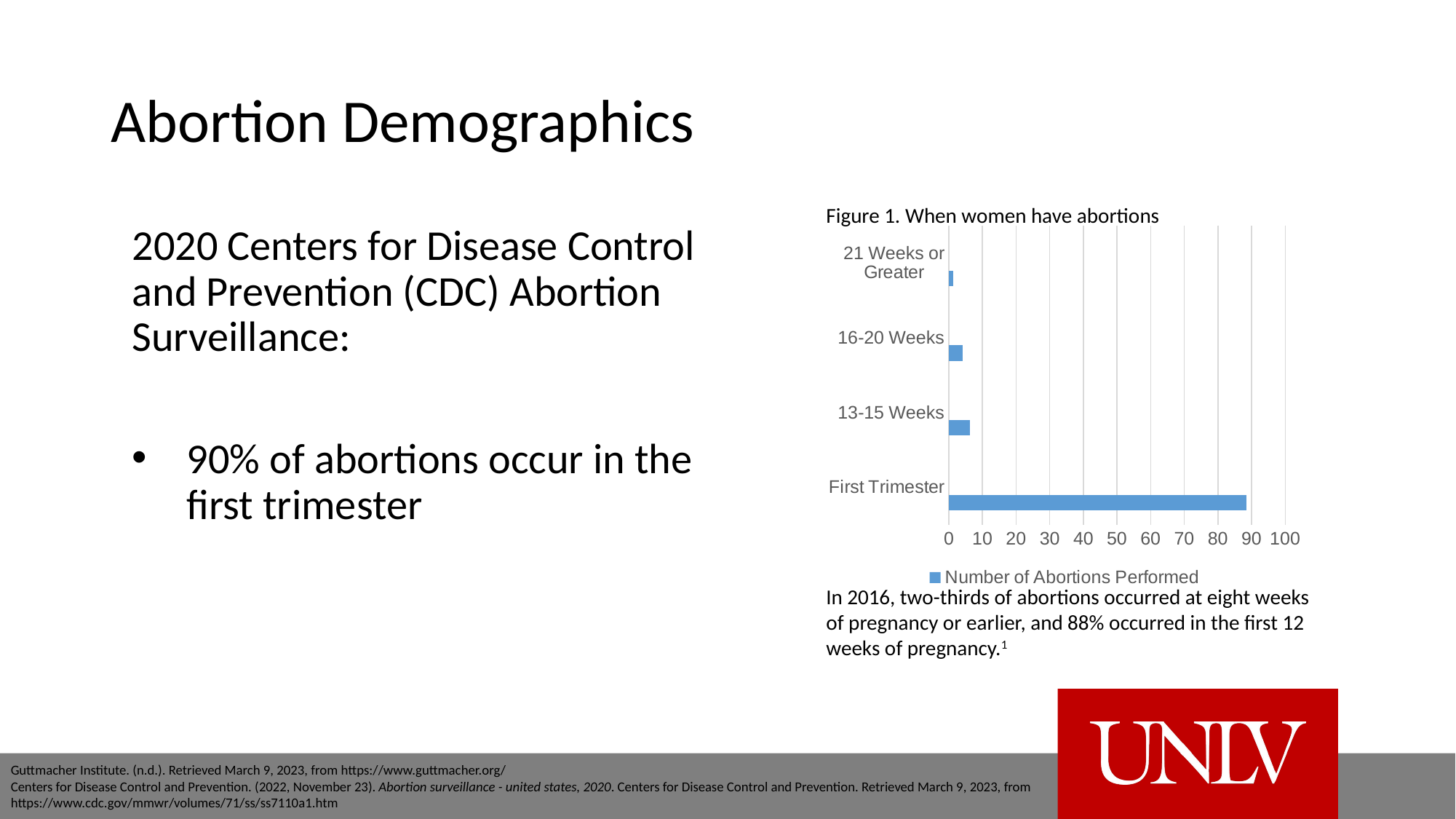

# Abortion Demographics
Figure 1. When women have abortions
In 2016, two-thirds of abortions occurred at eight weeks of pregnancy or earlier, and 88% occurred in the first 12 weeks of pregnancy.1
2020 Centers for Disease Control and Prevention (CDC) Abortion Surveillance:
90% of abortions occur in the first trimester
### Chart
| Category | Number of Abortions Performed | Column1 | Column2 |
|---|---|---|---|
| First Trimester | 88.5 | None | None |
| 13-15 Weeks | 6.2 | None | None |
| 16-20 Weeks | 4.0 | None | None |
| 21 Weeks or Greater | 1.3 | None | None |Guttmacher Institute. (n.d.). Retrieved March 9, 2023, from https://www.guttmacher.org/
Centers for Disease Control and Prevention. (2022, November 23). Abortion surveillance - united states, 2020. Centers for Disease Control and Prevention. Retrieved March 9, 2023, from https://www.cdc.gov/mmwr/volumes/71/ss/ss7110a1.htm

## Slide 4
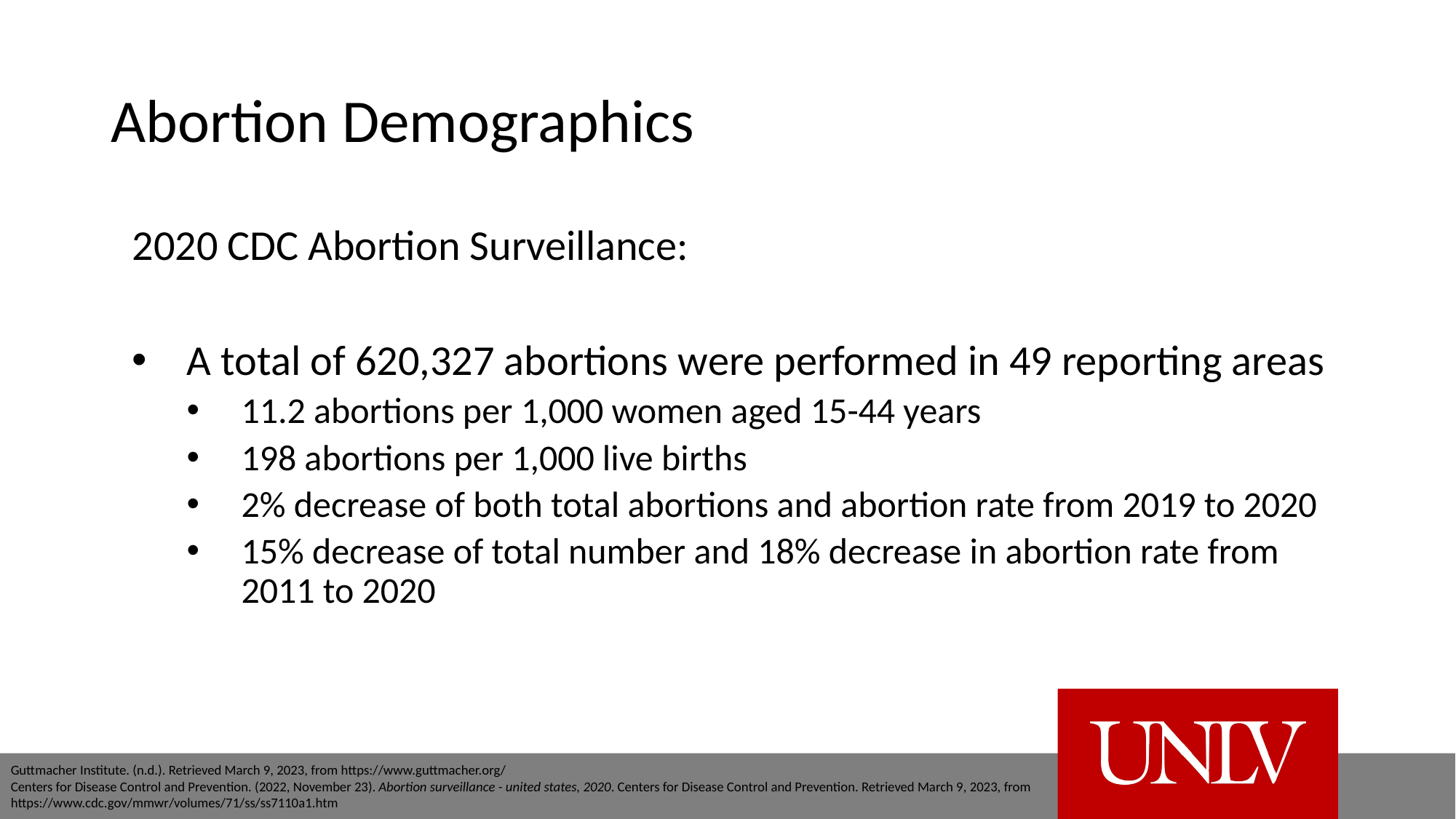

# Abortion Demographics
2020 CDC Abortion Surveillance:
A total of 620,327 abortions were performed in 49 reporting areas
11.2 abortions per 1,000 women aged 15-44 years
198 abortions per 1,000 live births
2% decrease of both total abortions and abortion rate from 2019 to 2020
15% decrease of total number and 18% decrease in abortion rate from 2011 to 2020
Guttmacher Institute. (n.d.). Retrieved March 9, 2023, from https://www.guttmacher.org/
Centers for Disease Control and Prevention. (2022, November 23). Abortion surveillance - united states, 2020. Centers for Disease Control and Prevention. Retrieved March 9, 2023, from https://www.cdc.gov/mmwr/volumes/71/ss/ss7110a1.htm

## Slide 5
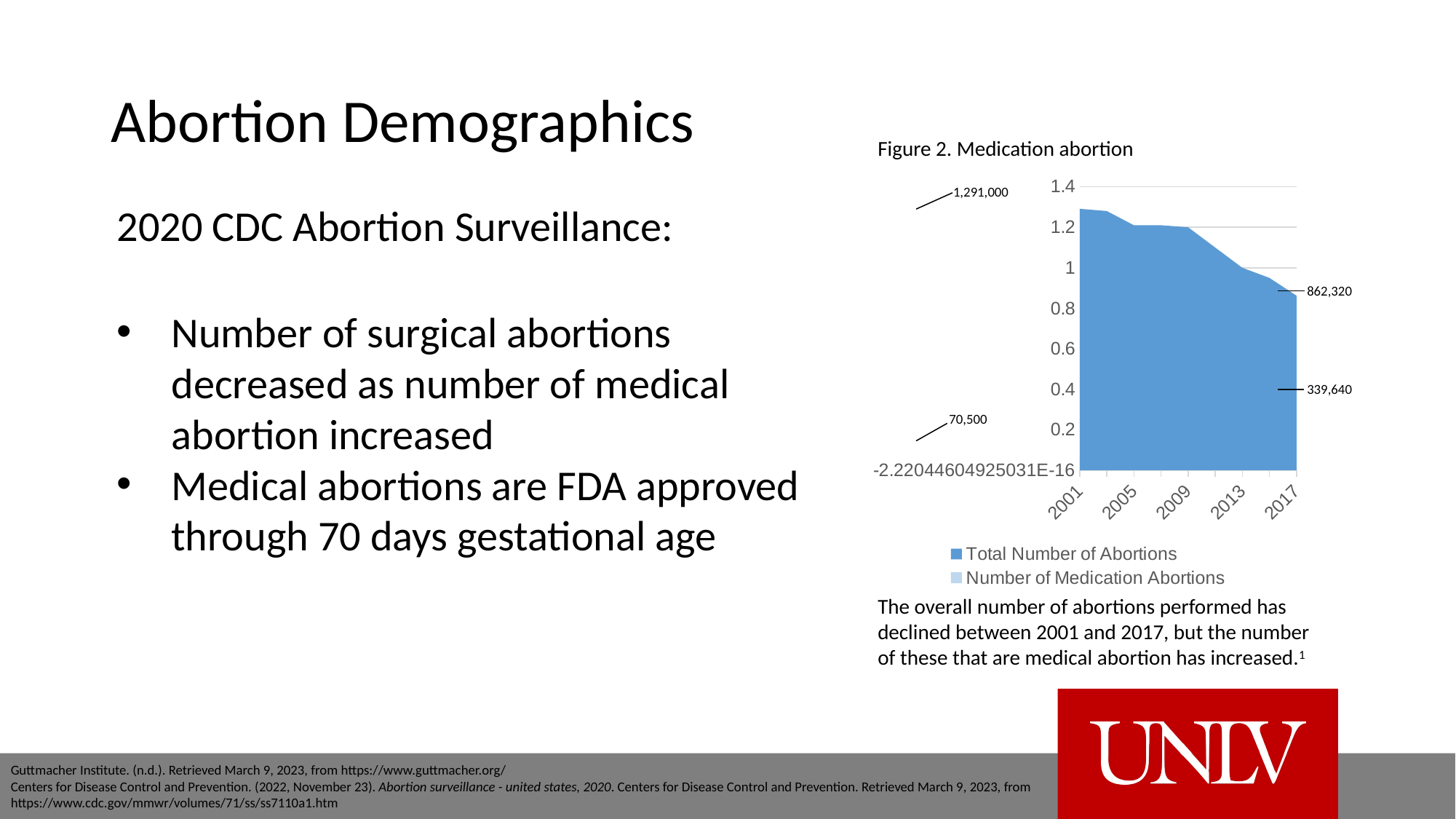

# Abortion Demographics
Figure 2. Medication abortion
The overall number of abortions performed has declined between 2001 and 2017, but the number of these that are medical abortion has increased.1
### Chart
| Category | Total Number of Abortions | Number of Medication Abortions |
|---|---|---|
| 2001 | 1.291 | 0.0705 |
| 2003 | 1.28 | 0.1 |
| 2005 | 1.21 | 0.15 |
| 2007 | 1.21 | 0.195 |
| 2009 | 1.2 | 0.21 |
| 2011 | 1.1 | 0.22 |
| 2013 | 1.0 | 0.25 |
| 2015 | 0.95 | 0.295 |
| 2017 | 0.86232 | 0.33964 |1,291,000
2020 CDC Abortion Surveillance:
Number of surgical abortions decreased as number of medical abortion increased
Medical abortions are FDA approved through 70 days gestational age
862,320
339,640
70,500
Guttmacher Institute. (n.d.). Retrieved March 9, 2023, from https://www.guttmacher.org/
Centers for Disease Control and Prevention. (2022, November 23). Abortion surveillance - united states, 2020. Centers for Disease Control and Prevention. Retrieved March 9, 2023, from https://www.cdc.gov/mmwr/volumes/71/ss/ss7110a1.htm

## Slide 6
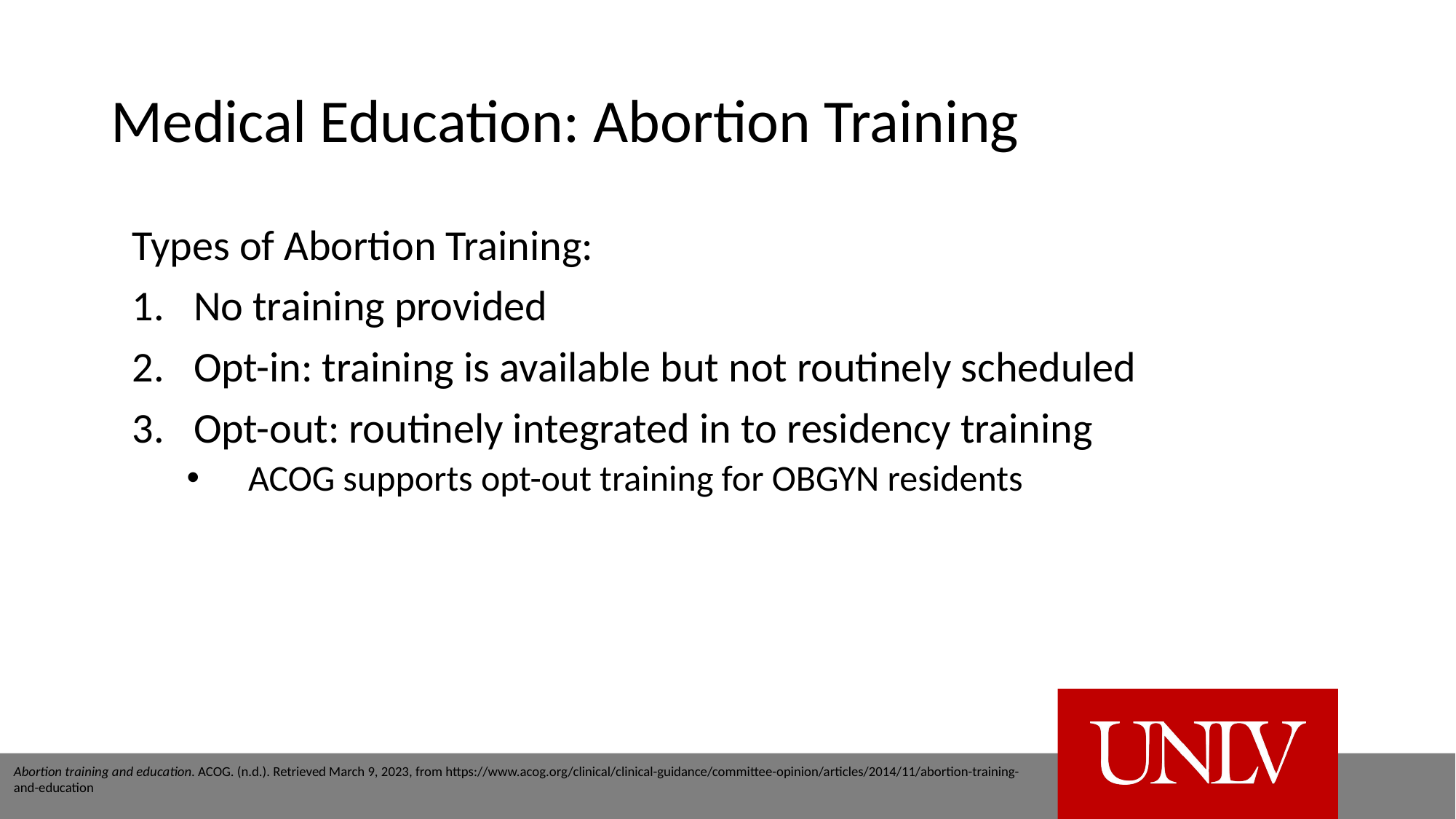

# Medical Education: Abortion Training
Types of Abortion Training:
No training provided
Opt-in: training is available but not routinely scheduled
Opt-out: routinely integrated in to residency training
ACOG supports opt-out training for OBGYN residents
Abortion training and education. ACOG. (n.d.). Retrieved March 9, 2023, from https://www.acog.org/clinical/clinical-guidance/committee-opinion/articles/2014/11/abortion-training-and-education

## Slide 7
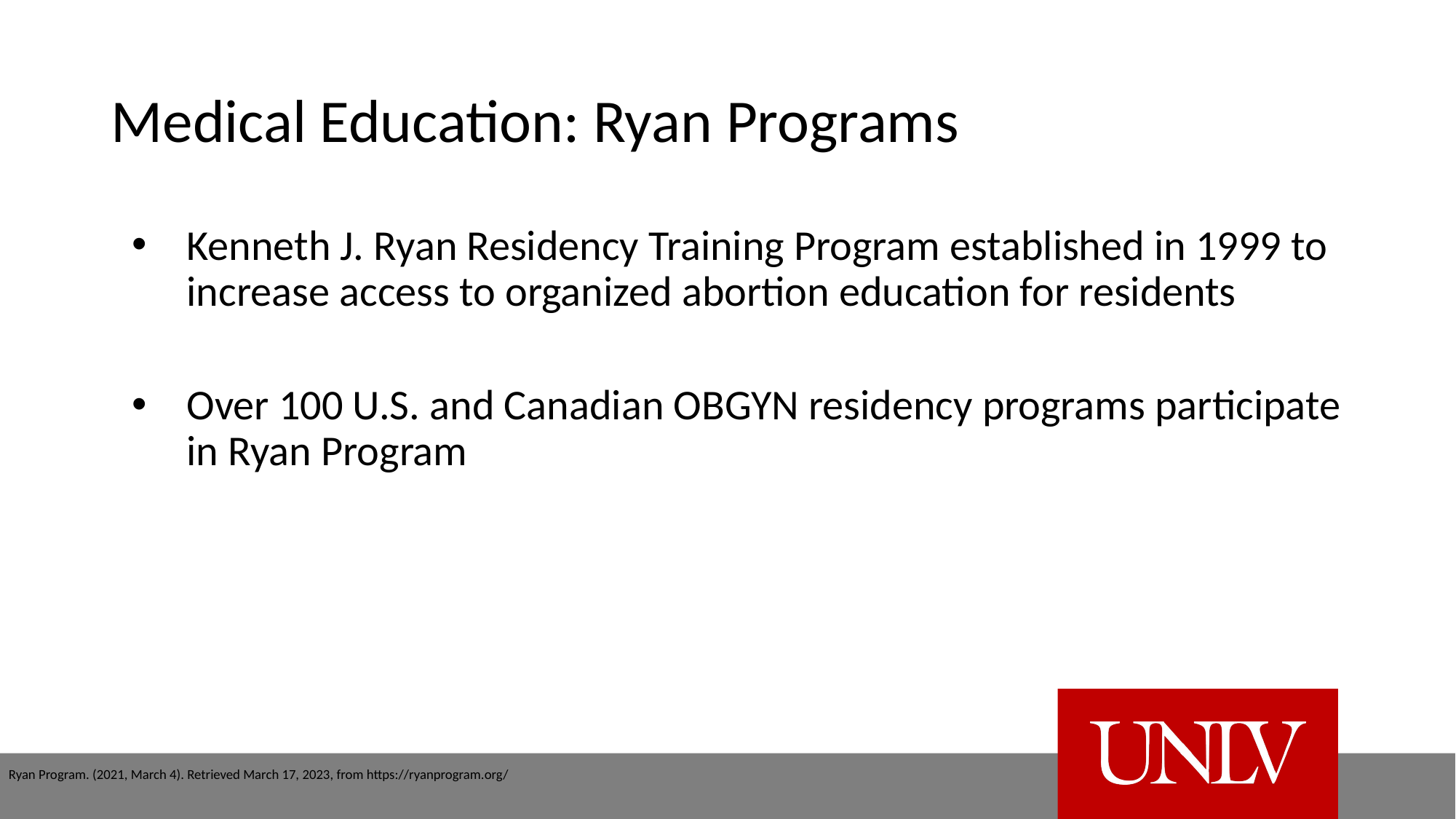

# Medical Education: Ryan Programs
Kenneth J. Ryan Residency Training Program established in 1999 to increase access to organized abortion education for residents
Over 100 U.S. and Canadian OBGYN residency programs participate in Ryan Program
Ryan Program. (2021, March 4). Retrieved March 17, 2023, from https://ryanprogram.org/

## Slide 8
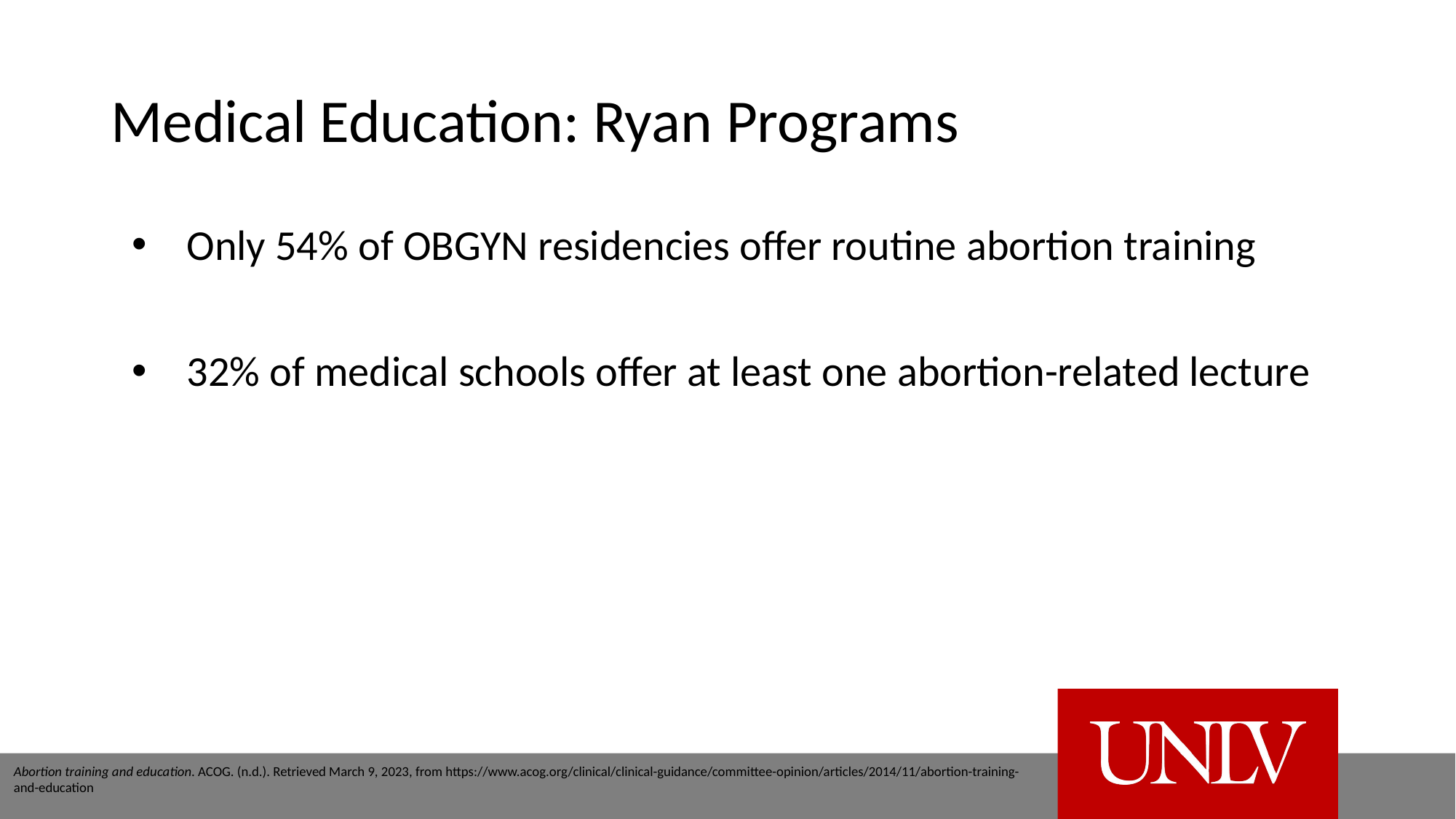

# Medical Education: Ryan Programs
Only 54% of OBGYN residencies offer routine abortion training
32% of medical schools offer at least one abortion-related lecture
Abortion training and education. ACOG. (n.d.). Retrieved March 9, 2023, from https://www.acog.org/clinical/clinical-guidance/committee-opinion/articles/2014/11/abortion-training-and-education

## Slide 9
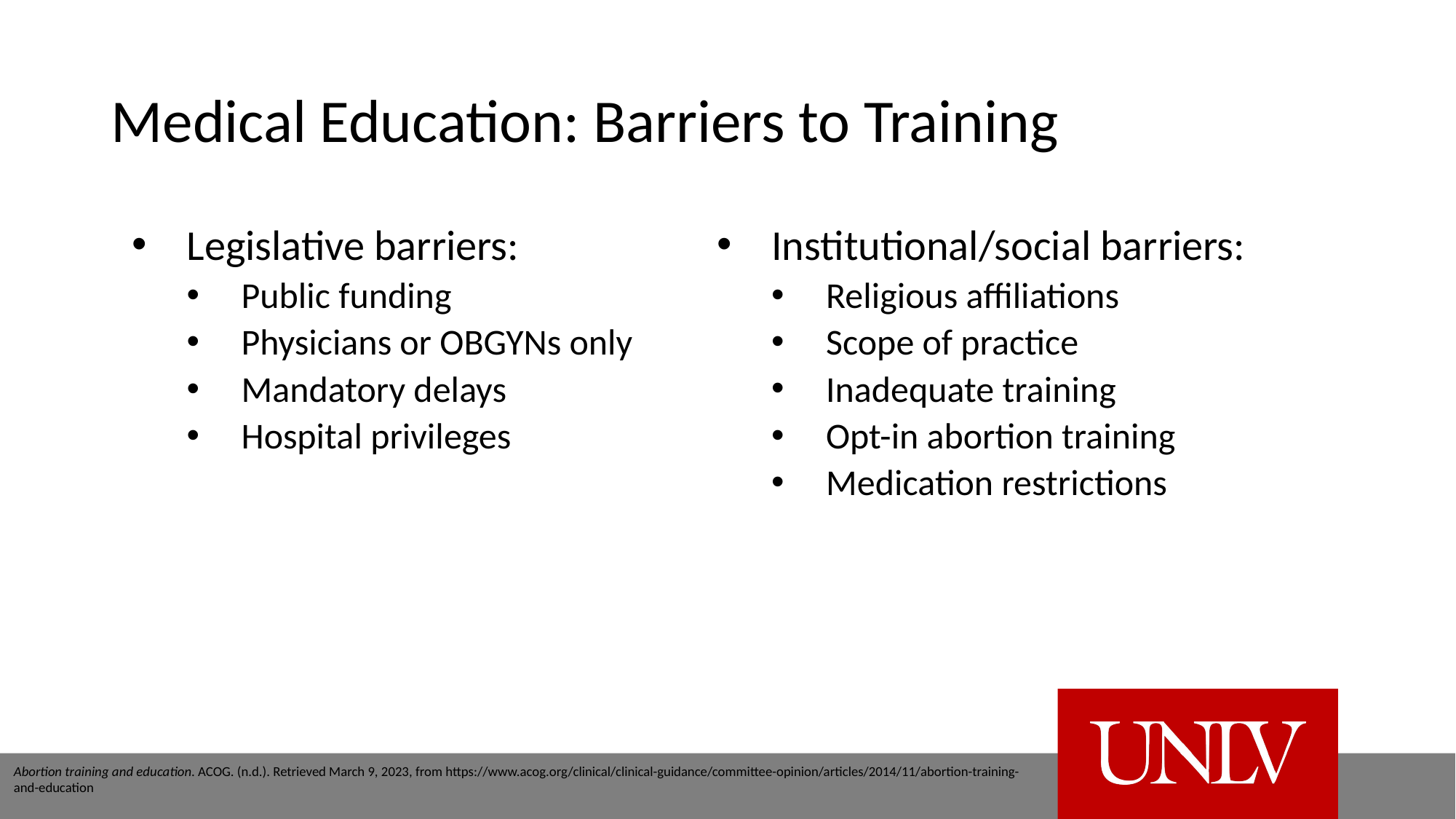

# Medical Education: Barriers to Training
Legislative barriers:
Public funding
Physicians or OBGYNs only
Mandatory delays
Hospital privileges
Institutional/social barriers:
Religious affiliations
Scope of practice
Inadequate training
Opt-in abortion training
Medication restrictions
Abortion training and education. ACOG. (n.d.). Retrieved March 9, 2023, from https://www.acog.org/clinical/clinical-guidance/committee-opinion/articles/2014/11/abortion-training-and-education

## Slide 10
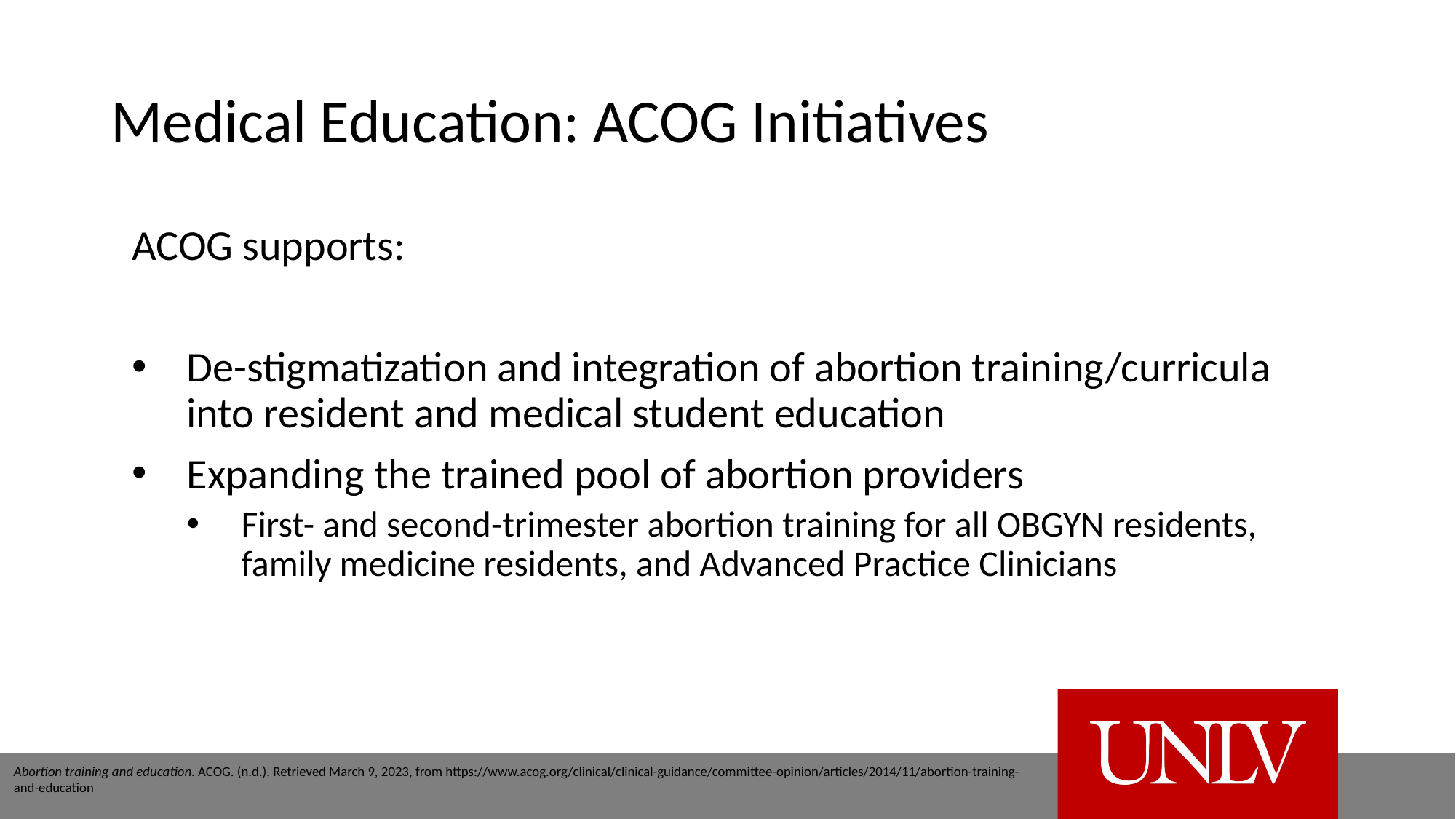

# Medical Education: ACOG Initiatives
ACOG supports:
De-stigmatization and integration of abortion training/curricula into resident and medical student education
Expanding the trained pool of abortion providers
First- and second-trimester abortion training for all OBGYN residents, family medicine residents, and Advanced Practice Clinicians
Abortion training and education. ACOG. (n.d.). Retrieved March 9, 2023, from https://www.acog.org/clinical/clinical-guidance/committee-opinion/articles/2014/11/abortion-training-and-education

## Slide 11
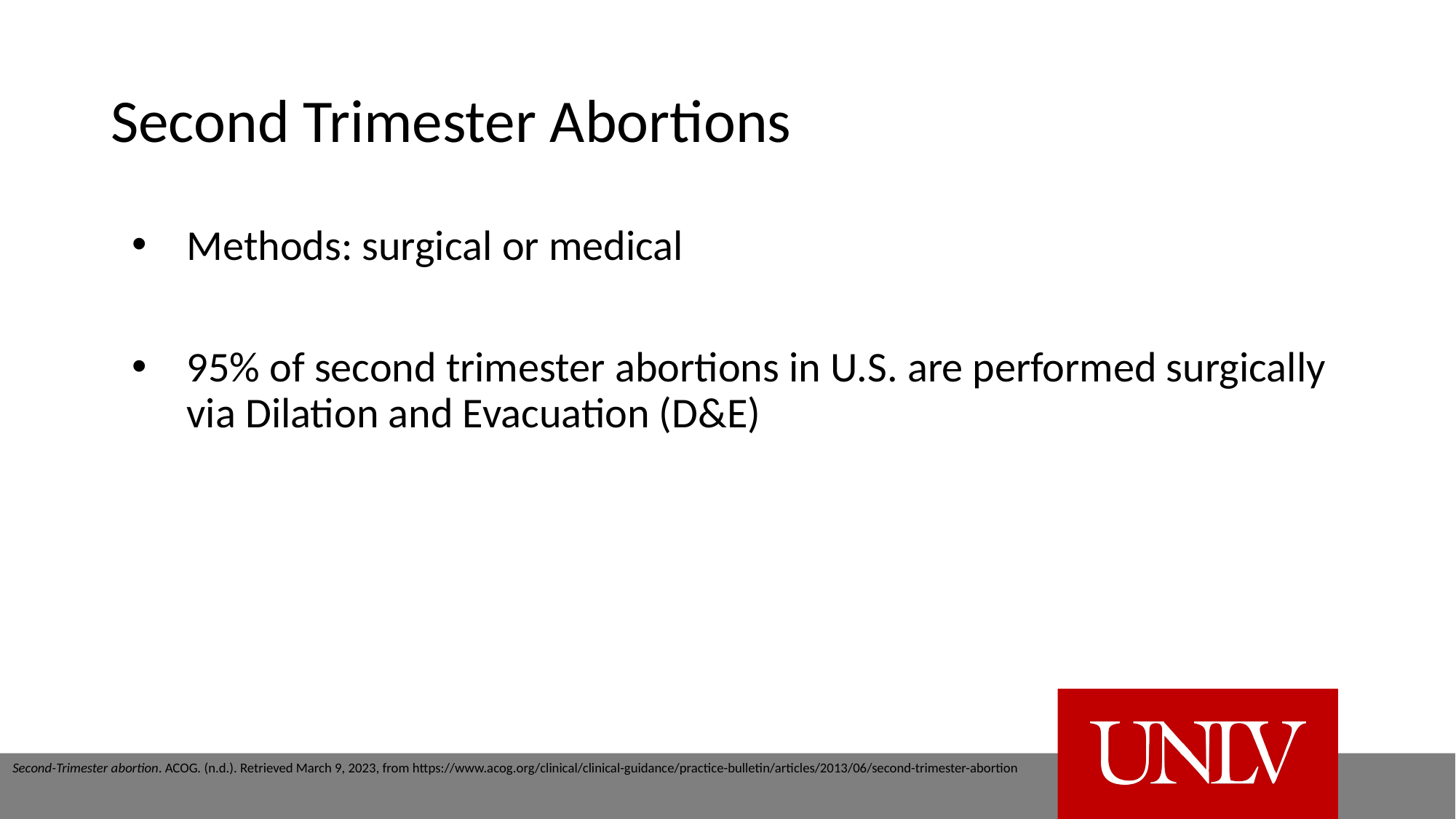

# Second Trimester Abortions
Methods: surgical or medical
95% of second trimester abortions in U.S. are performed surgically via Dilation and Evacuation (D&E)
Second-Trimester abortion. ACOG. (n.d.). Retrieved March 9, 2023, from https://www.acog.org/clinical/clinical-guidance/practice-bulletin/articles/2013/06/second-trimester-abortion

## Slide 12
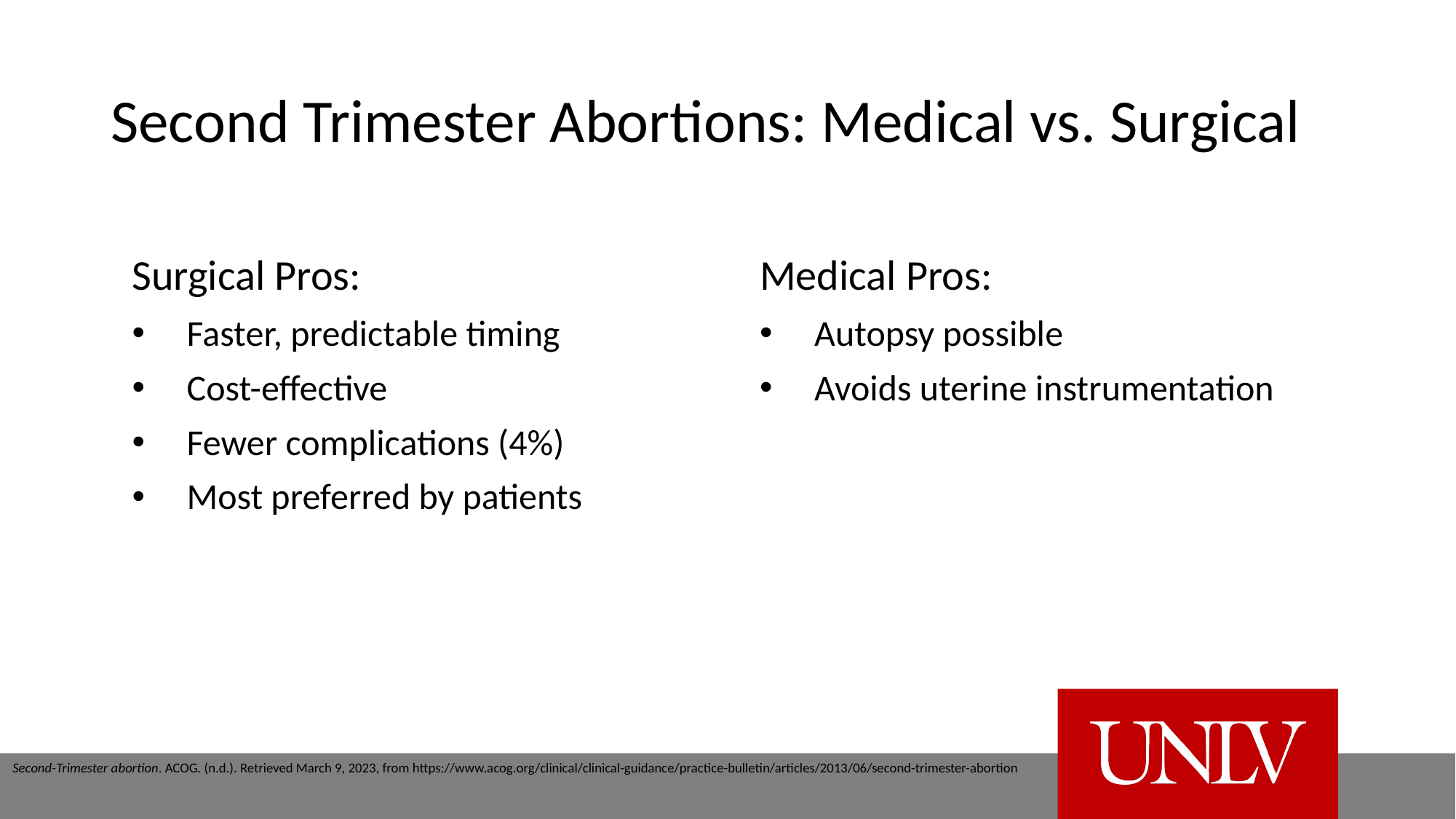

# Second Trimester Abortions: Medical vs. Surgical
Surgical Pros:
Faster, predictable timing
Cost-effective
Fewer complications (4%)
Most preferred by patients
Medical Pros:
Autopsy possible
Avoids uterine instrumentation
Second-Trimester abortion. ACOG. (n.d.). Retrieved March 9, 2023, from https://www.acog.org/clinical/clinical-guidance/practice-bulletin/articles/2013/06/second-trimester-abortion

## Slide 13
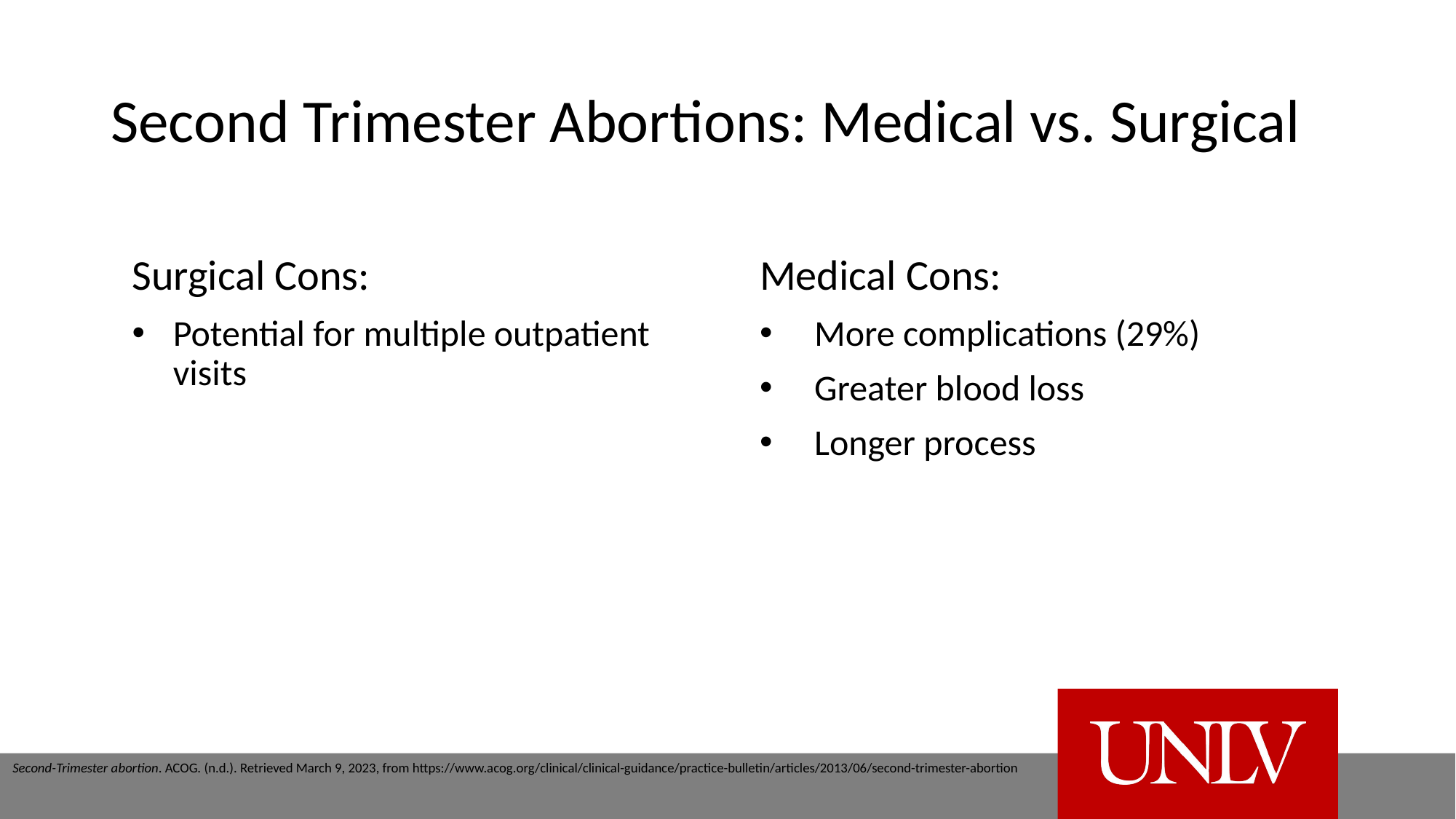

# Second Trimester Abortions: Medical vs. Surgical
Surgical Cons:
Potential for multiple outpatient visits
Medical Cons:
More complications (29%)
Greater blood loss
Longer process
Second-Trimester abortion. ACOG. (n.d.). Retrieved March 9, 2023, from https://www.acog.org/clinical/clinical-guidance/practice-bulletin/articles/2013/06/second-trimester-abortion

## Slide 14
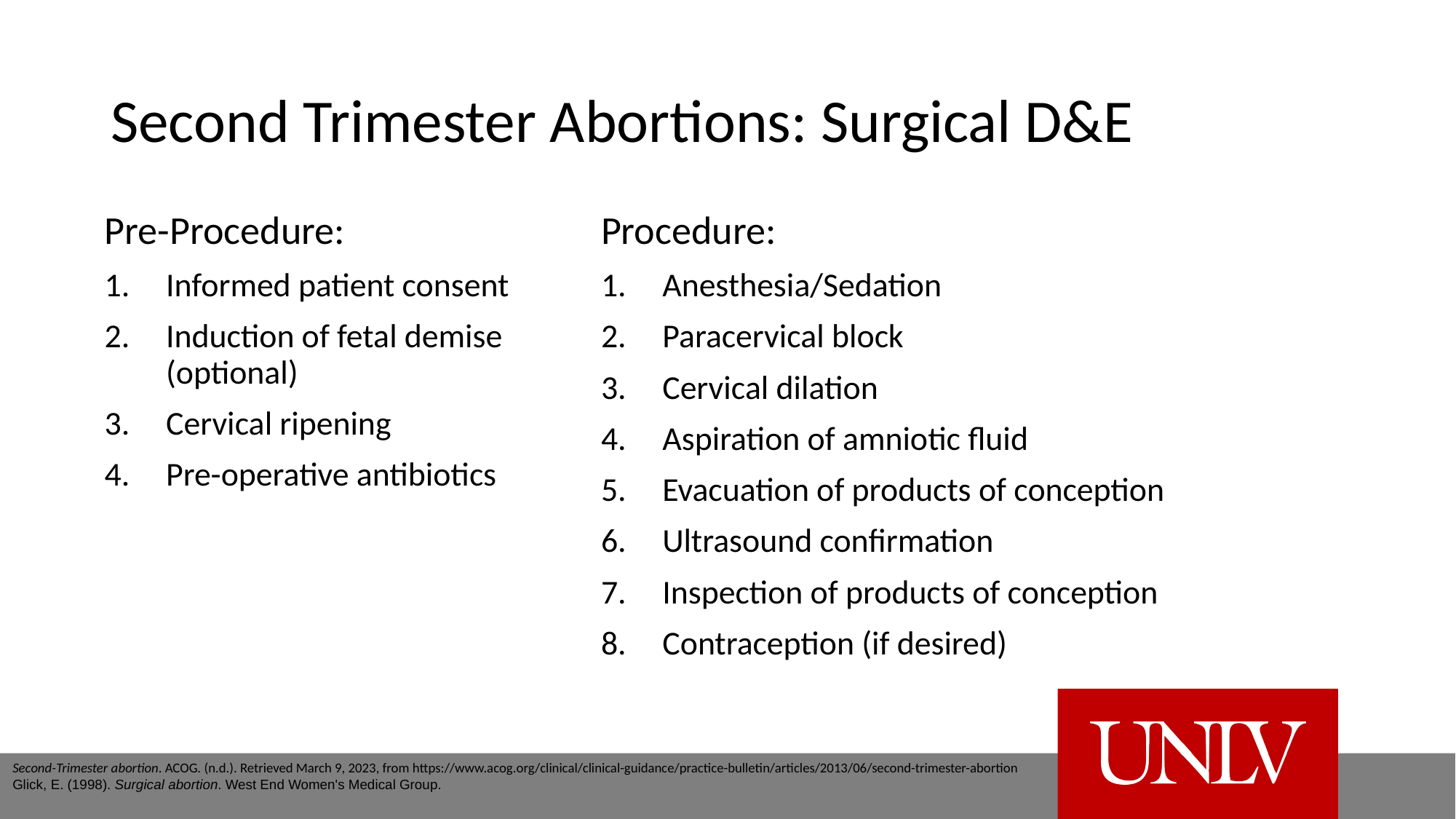

# Second Trimester Abortions: Surgical D&E
Pre-Procedure:
Informed patient consent
Induction of fetal demise (optional)
Cervical ripening
Pre-operative antibiotics
Procedure:
Anesthesia/Sedation
Paracervical block
Cervical dilation
Aspiration of amniotic fluid
Evacuation of products of conception
Ultrasound confirmation
Inspection of products of conception
Contraception (if desired)
Second-Trimester abortion. ACOG. (n.d.). Retrieved March 9, 2023, from https://www.acog.org/clinical/clinical-guidance/practice-bulletin/articles/2013/06/second-trimester-abortion
Glick, E. (1998). Surgical abortion. West End Women's Medical Group.

## Slide 15
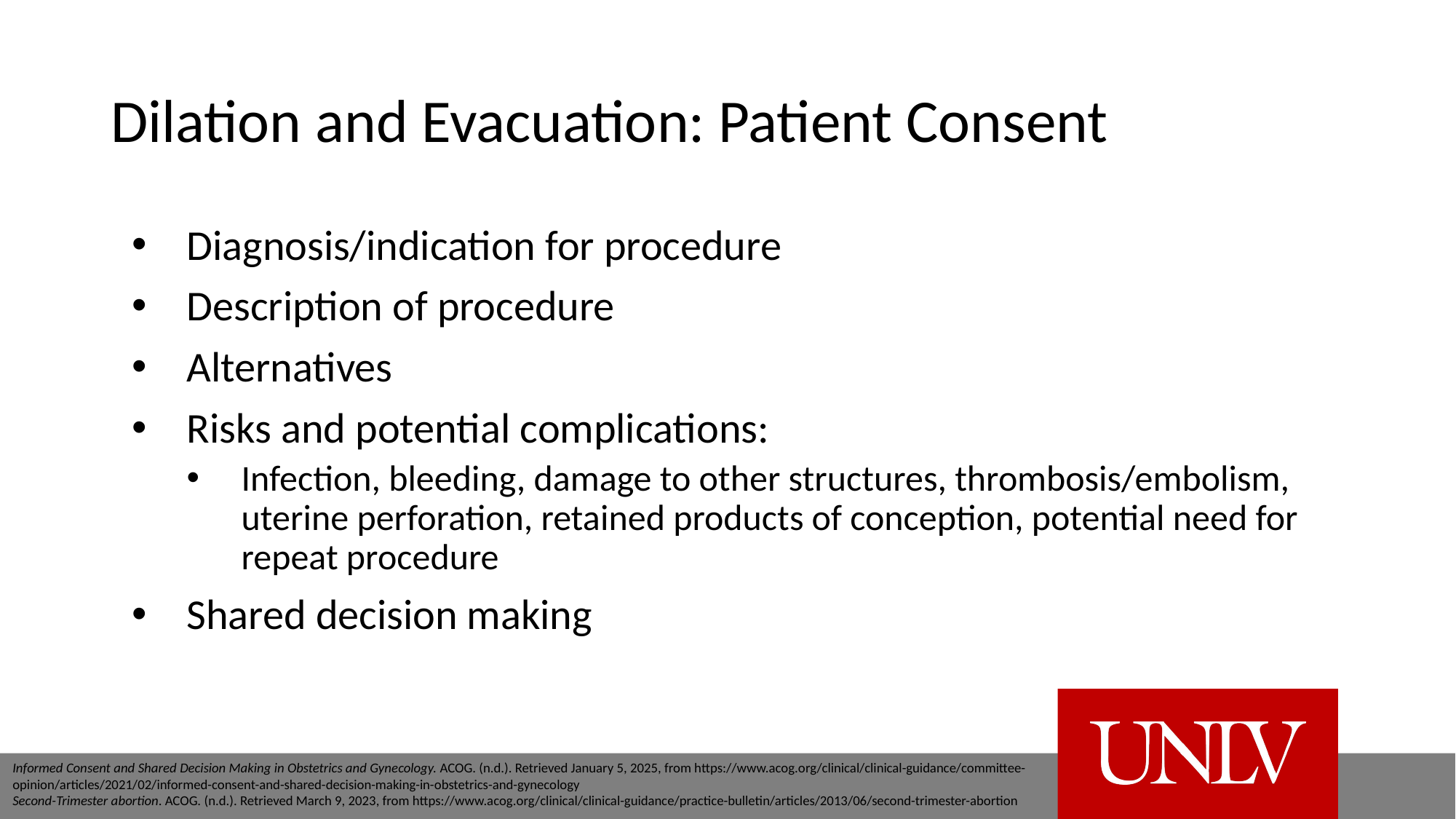

# Dilation and Evacuation: Patient Consent
Diagnosis/indication for procedure
Description of procedure
Alternatives
Risks and potential complications:
Infection, bleeding, damage to other structures, thrombosis/embolism, uterine perforation, retained products of conception, potential need for repeat procedure
Shared decision making
Informed Consent and Shared Decision Making in Obstetrics and Gynecology. ACOG. (n.d.). Retrieved January 5, 2025, from https://www.acog.org/clinical/clinical-guidance/committee-opinion/articles/2021/02/informed-consent-and-shared-decision-making-in-obstetrics-and-gynecology
Second-Trimester abortion. ACOG. (n.d.). Retrieved March 9, 2023, from https://www.acog.org/clinical/clinical-guidance/practice-bulletin/articles/2013/06/second-trimester-abortion

## Slide 16
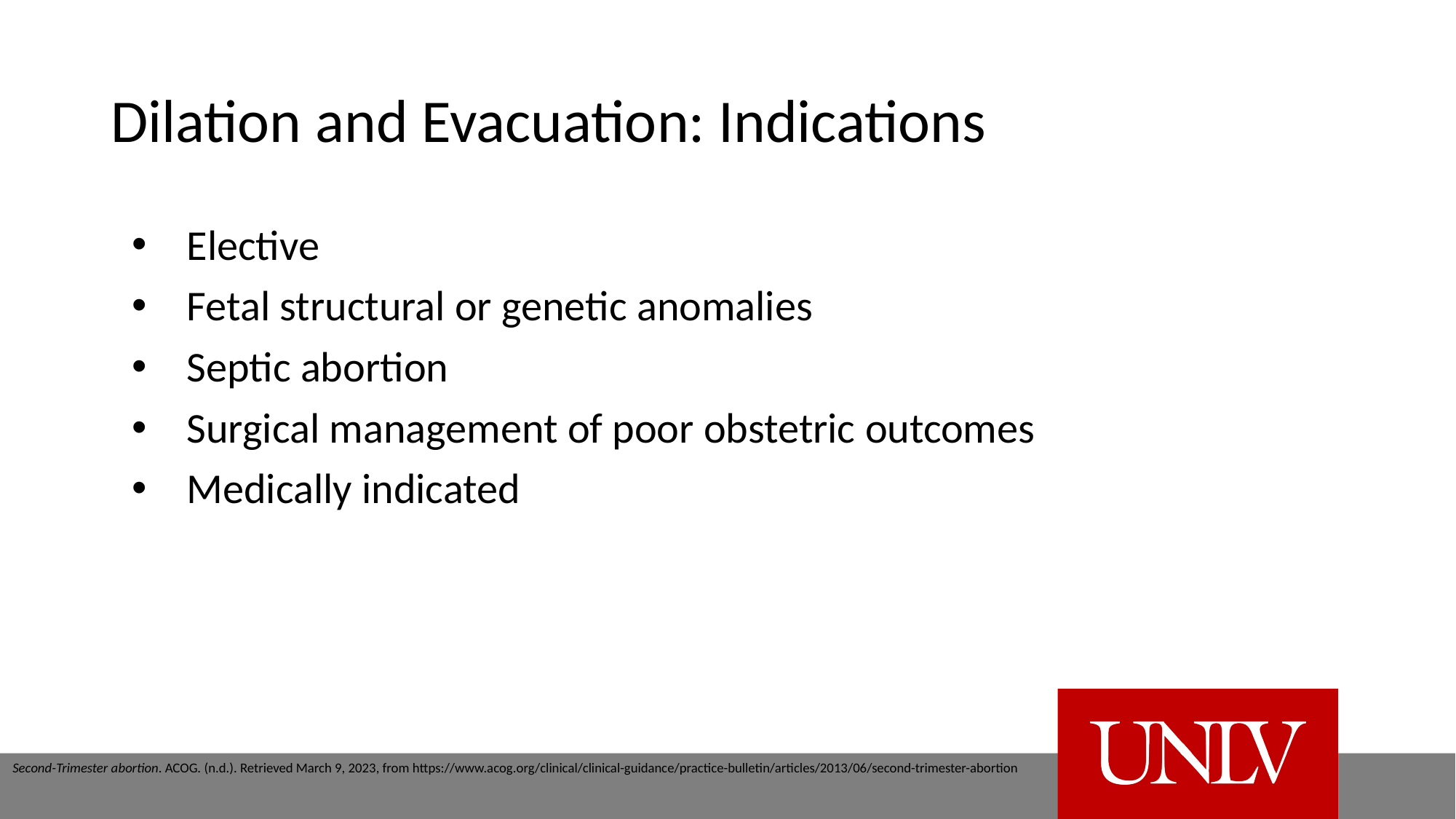

# Dilation and Evacuation: Indications
Elective
Fetal structural or genetic anomalies
Septic abortion
Surgical management of poor obstetric outcomes
Medically indicated
Second-Trimester abortion. ACOG. (n.d.). Retrieved March 9, 2023, from https://www.acog.org/clinical/clinical-guidance/practice-bulletin/articles/2013/06/second-trimester-abortion

## Slide 17
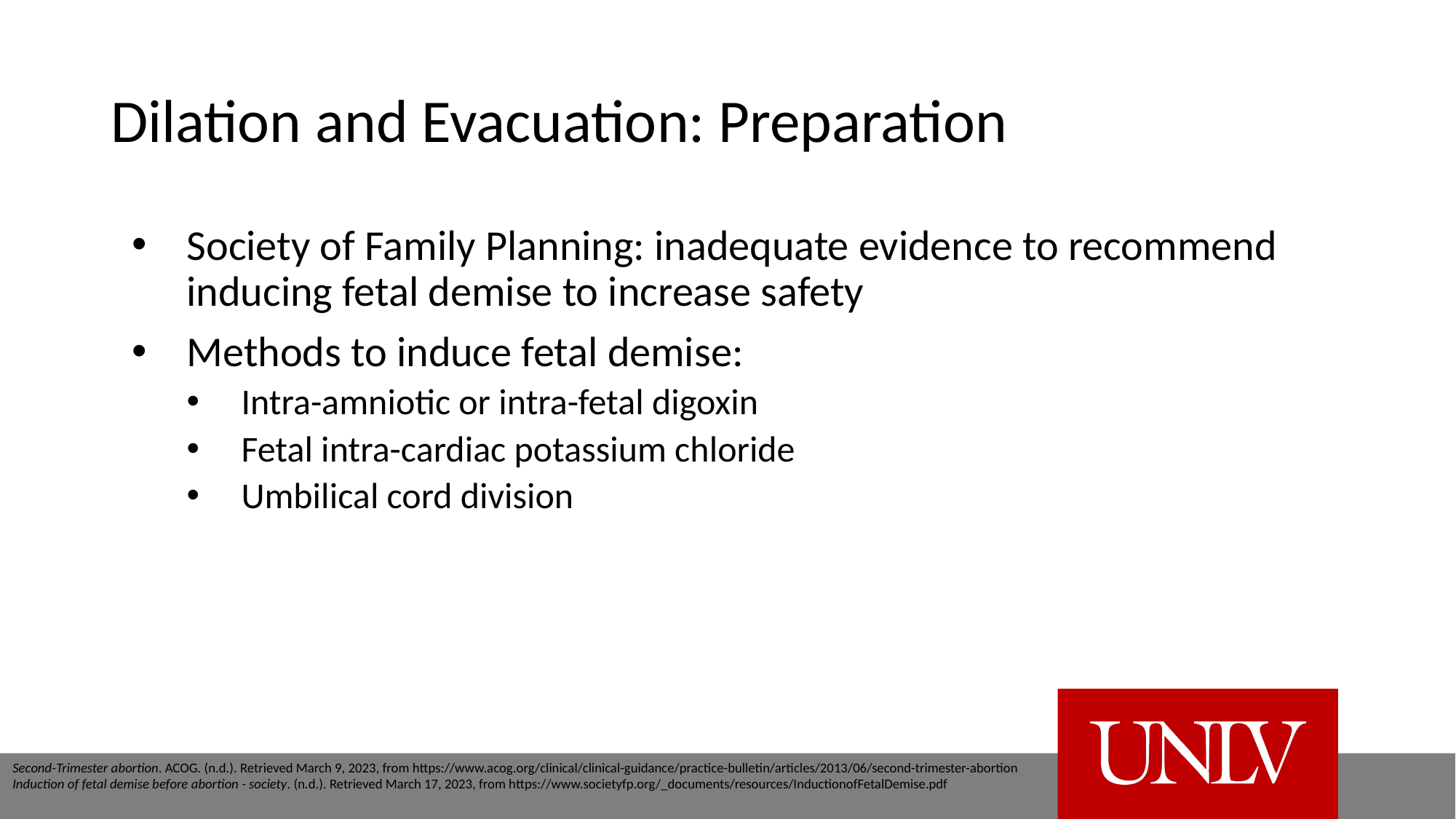

# Dilation and Evacuation: Preparation
Society of Family Planning: inadequate evidence to recommend inducing fetal demise to increase safety
Methods to induce fetal demise:
Intra-amniotic or intra-fetal digoxin
Fetal intra-cardiac potassium chloride
Umbilical cord division
Second-Trimester abortion. ACOG. (n.d.). Retrieved March 9, 2023, from https://www.acog.org/clinical/clinical-guidance/practice-bulletin/articles/2013/06/second-trimester-abortion Induction of fetal demise before abortion - society. (n.d.). Retrieved March 17, 2023, from https://www.societyfp.org/_documents/resources/InductionofFetalDemise.pdf

## Slide 18
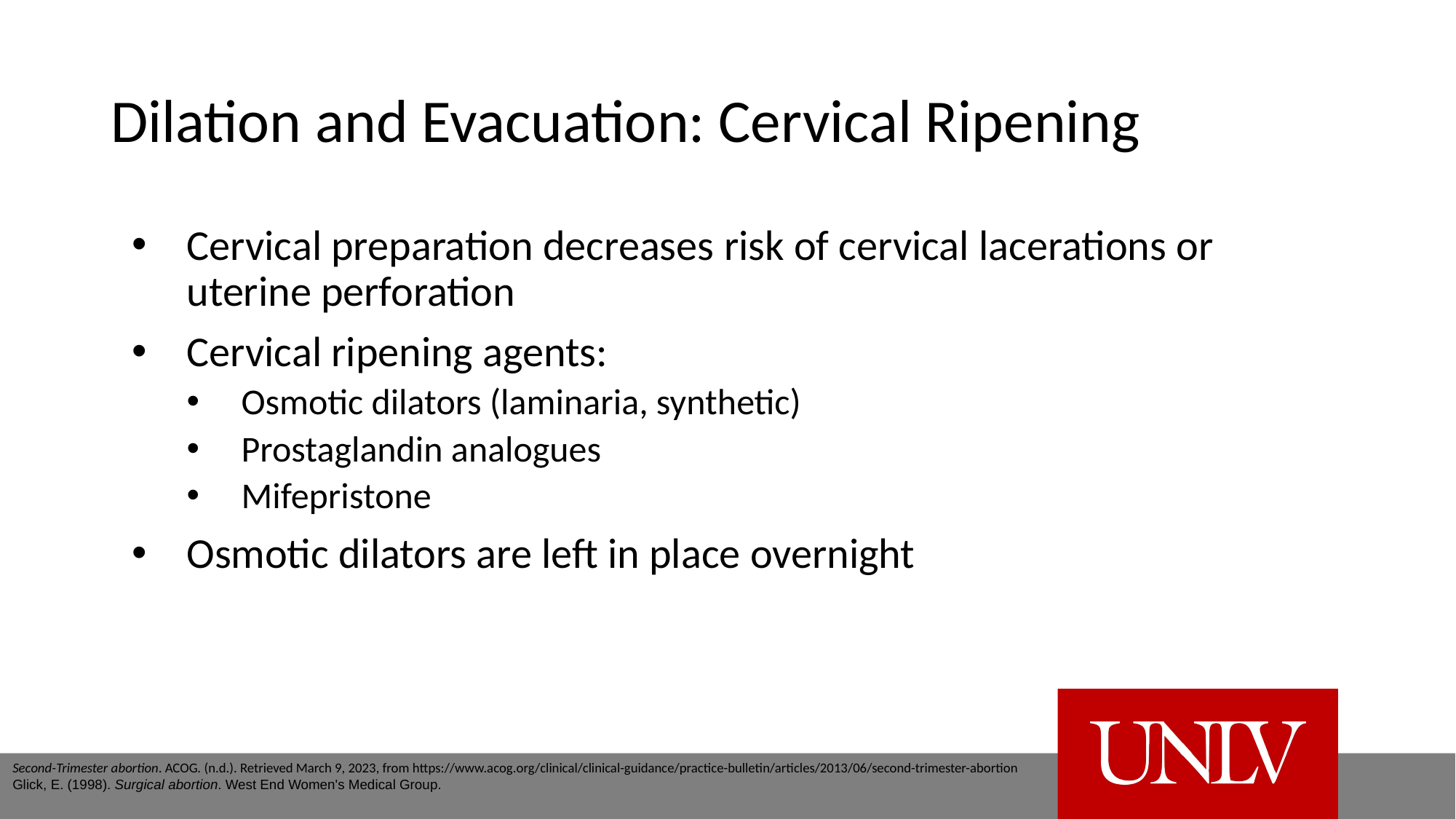

# Dilation and Evacuation: Cervical Ripening
Cervical preparation decreases risk of cervical lacerations or uterine perforation
Cervical ripening agents:
Osmotic dilators (laminaria, synthetic)
Prostaglandin analogues
Mifepristone
Osmotic dilators are left in place overnight
Second-Trimester abortion. ACOG. (n.d.). Retrieved March 9, 2023, from https://www.acog.org/clinical/clinical-guidance/practice-bulletin/articles/2013/06/second-trimester-abortion
Glick, E. (1998). Surgical abortion. West End Women's Medical Group.

## Slide 19
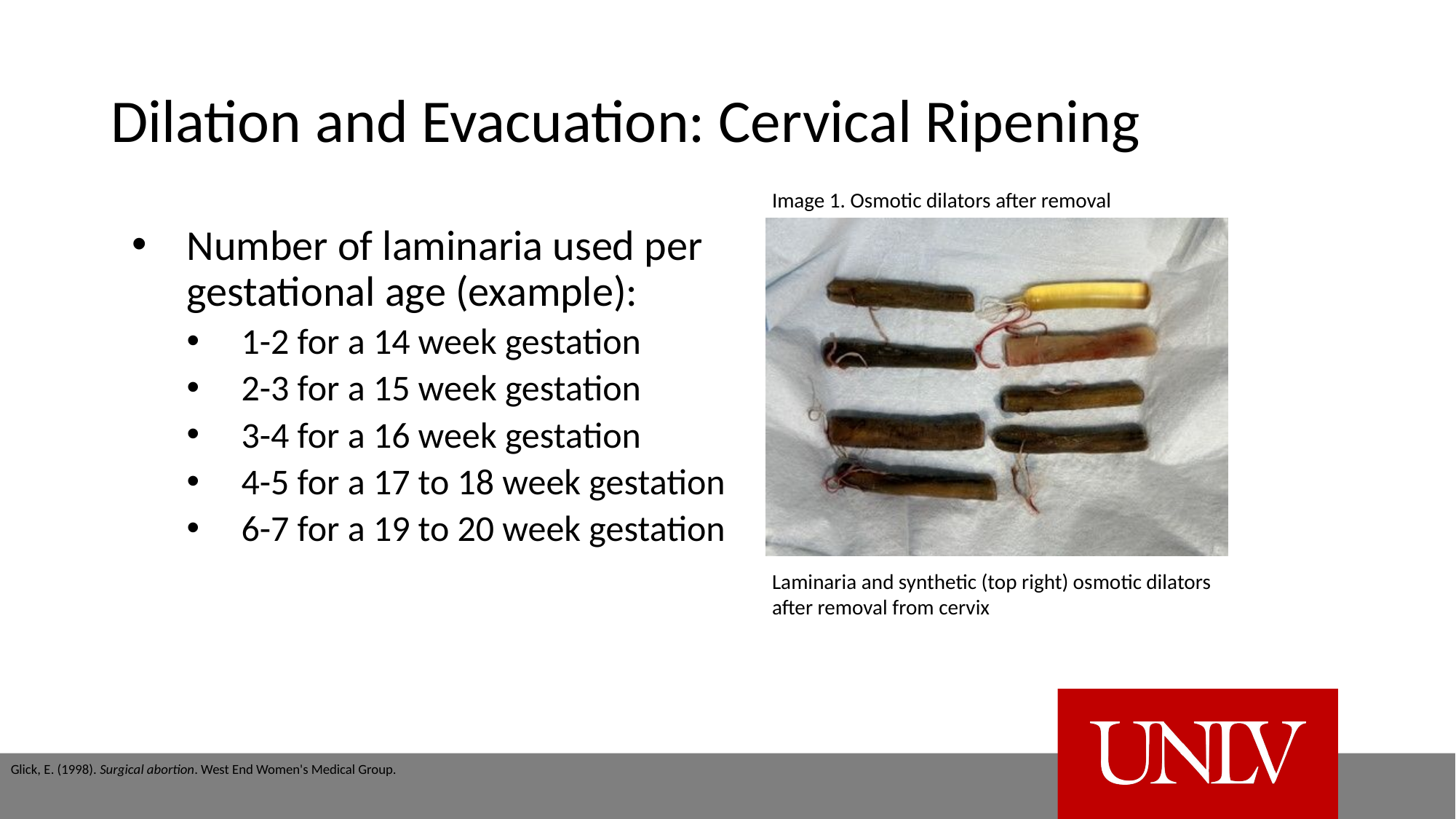

# Dilation and Evacuation: Cervical Ripening
Image 1. Osmotic dilators after removal
Laminaria and synthetic (top right) osmotic dilators after removal from cervix
Number of laminaria used per gestational age (example):
1-2 for a 14 week gestation
2-3 for a 15 week gestation
3-4 for a 16 week gestation
4-5 for a 17 to 18 week gestation
6-7 for a 19 to 20 week gestation
Glick, E. (1998). Surgical abortion. West End Women's Medical Group.

## Slide 20
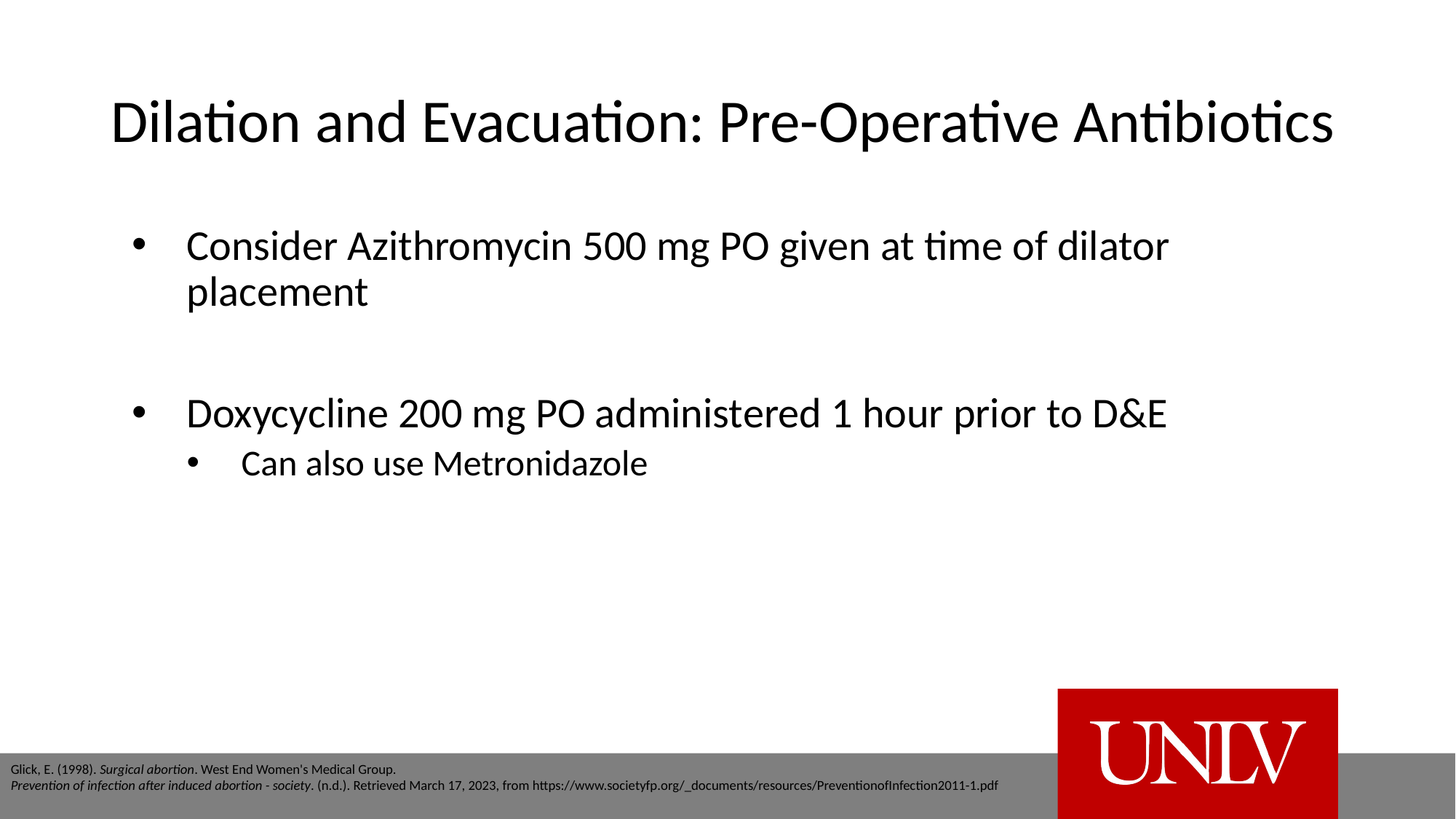

# Dilation and Evacuation: Pre-Operative Antibiotics
Consider Azithromycin 500 mg PO given at time of dilator placement
Doxycycline 200 mg PO administered 1 hour prior to D&E
Can also use Metronidazole
Glick, E. (1998). Surgical abortion. West End Women's Medical Group.
Prevention of infection after induced abortion - society. (n.d.). Retrieved March 17, 2023, from https://www.societyfp.org/_documents/resources/PreventionofInfection2011-1.pdf

## Slide 21
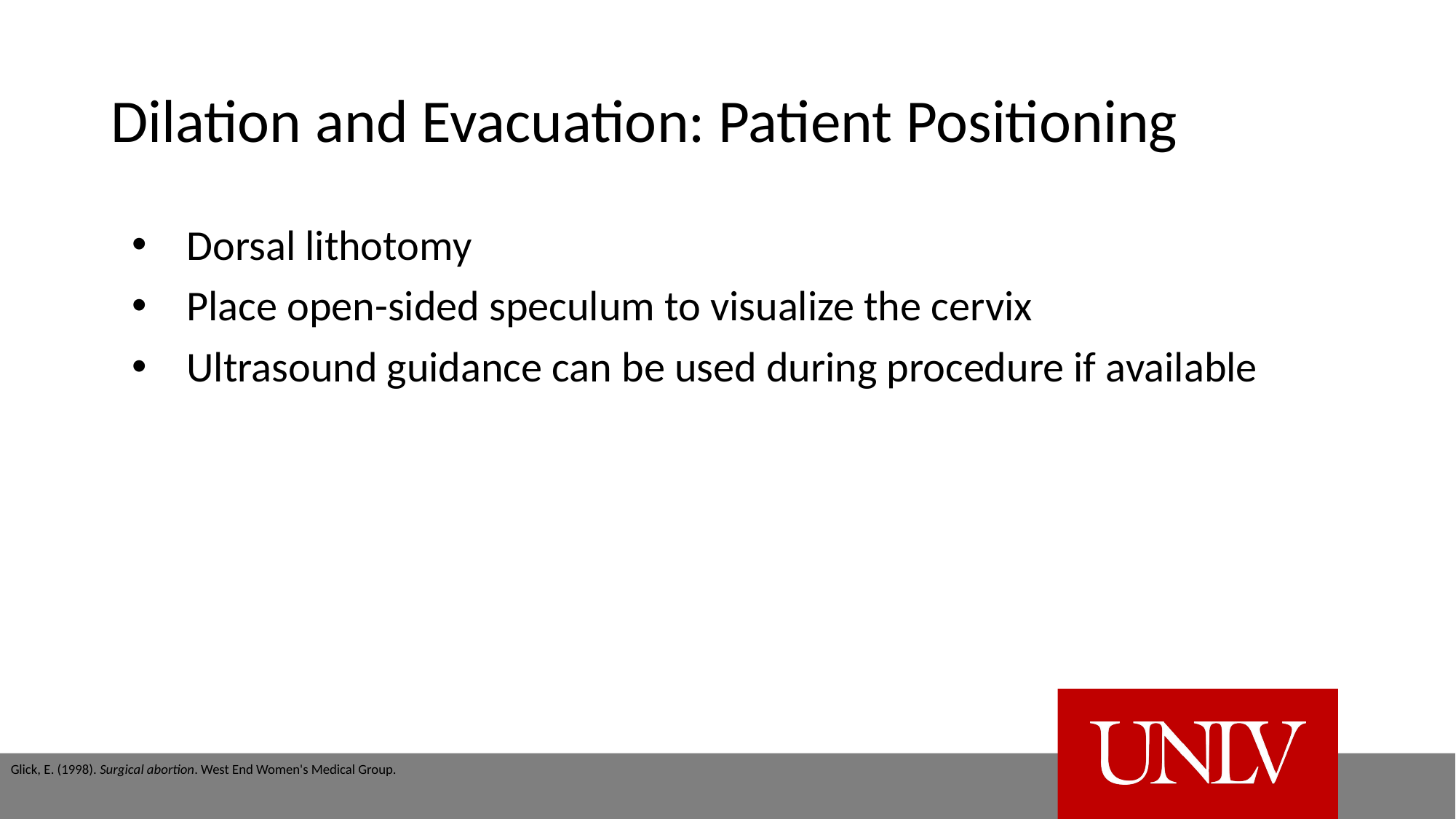

# Dilation and Evacuation: Patient Positioning
Dorsal lithotomy
Place open-sided speculum to visualize the cervix
Ultrasound guidance can be used during procedure if available
Glick, E. (1998). Surgical abortion. West End Women's Medical Group.

## Slide 22
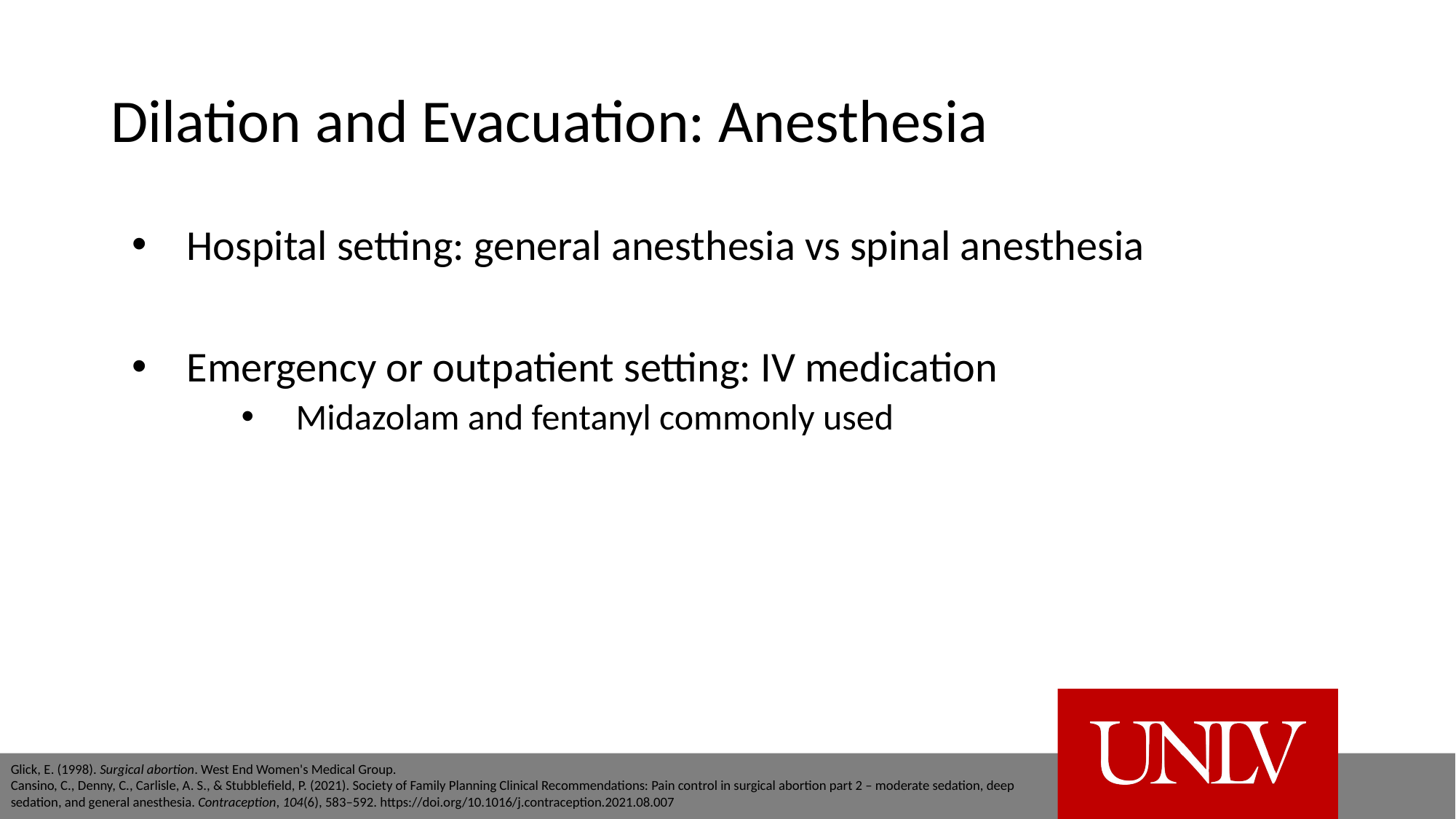

# Dilation and Evacuation: Anesthesia
Hospital setting: general anesthesia vs spinal anesthesia
Emergency or outpatient setting: IV medication
Midazolam and fentanyl commonly used
Glick, E. (1998). Surgical abortion. West End Women's Medical Group.
Cansino, C., Denny, C., Carlisle, A. S., & Stubblefield, P. (2021). Society of Family Planning Clinical Recommendations: Pain control in surgical abortion part 2 – moderate sedation, deep sedation, and general anesthesia. Contraception, 104(6), 583–592. https://doi.org/10.1016/j.contraception.2021.08.007

## Slide 23
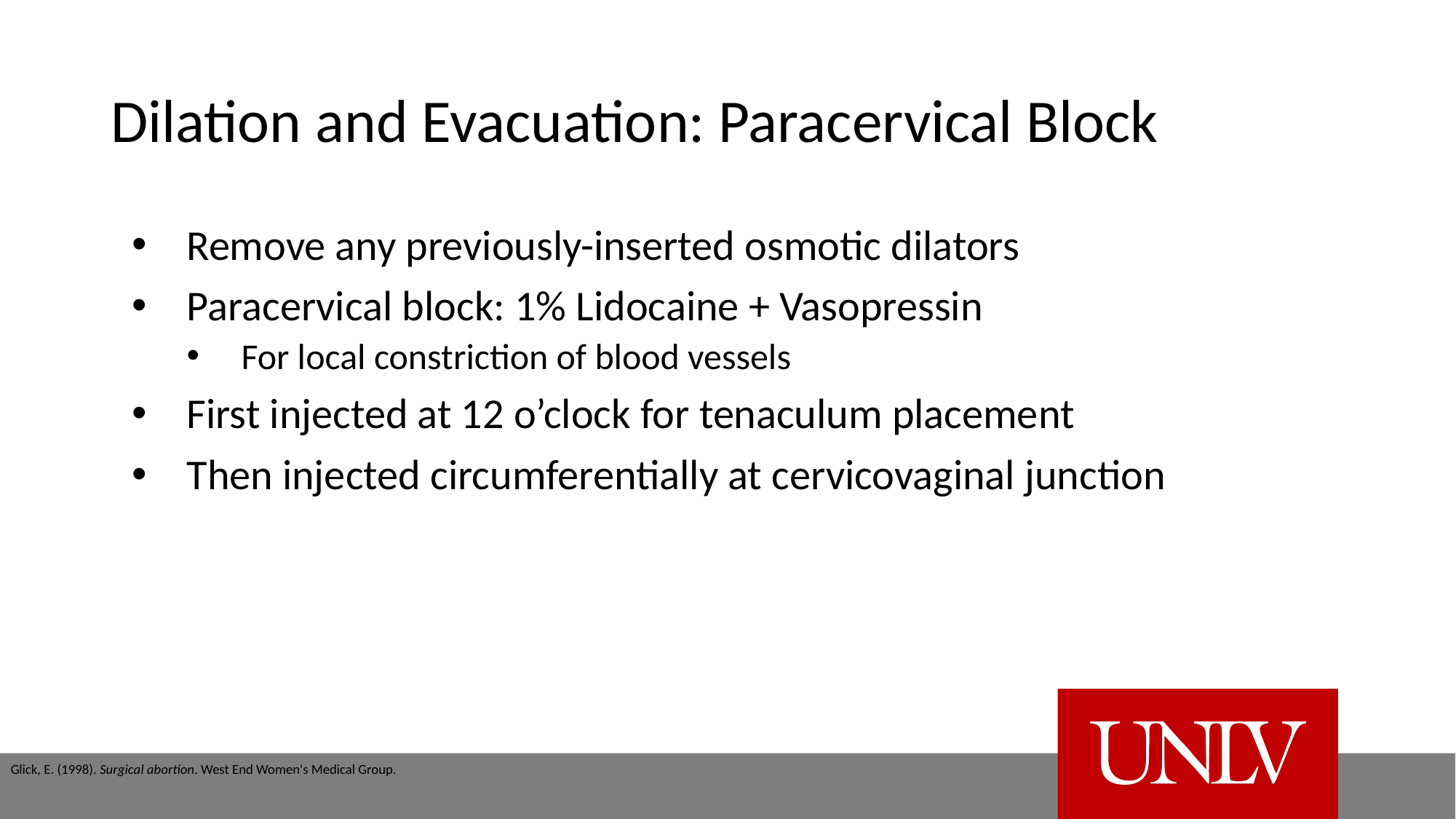

# Dilation and Evacuation: Paracervical Block
Remove any previously-inserted osmotic dilators
Paracervical block: 1% Lidocaine + Vasopressin
For local constriction of blood vessels
First injected at 12 o’clock for tenaculum placement
Then injected circumferentially at cervicovaginal junction
Glick, E. (1998). Surgical abortion. West End Women's Medical Group.

## Slide 24
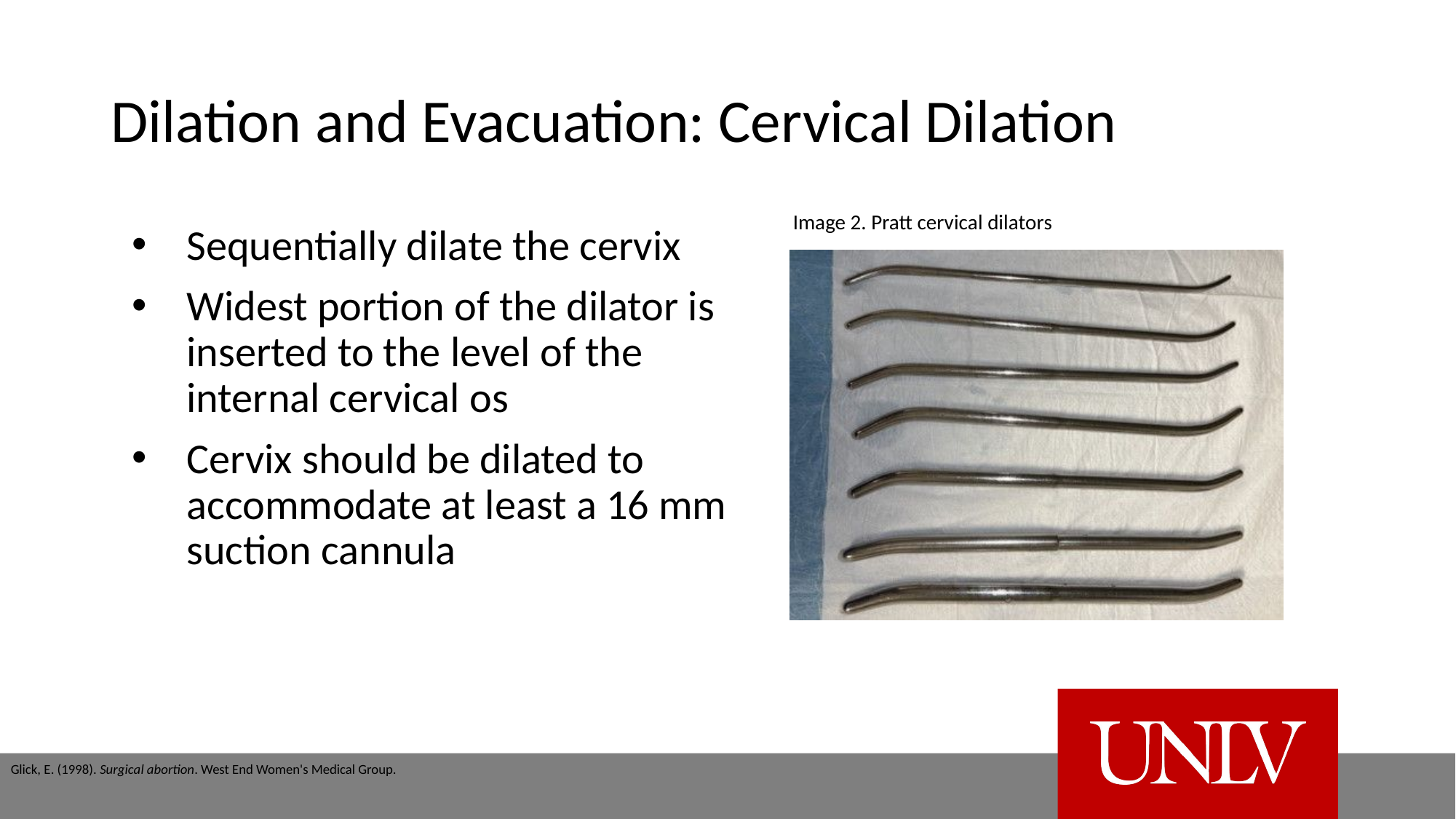

# Dilation and Evacuation: Cervical Dilation
Image 2. Pratt cervical dilators
Sequentially dilate the cervix
Widest portion of the dilator is inserted to the level of the internal cervical os
Cervix should be dilated to accommodate at least a 16 mm suction cannula
Glick, E. (1998). Surgical abortion. West End Women's Medical Group.

## Slide 25
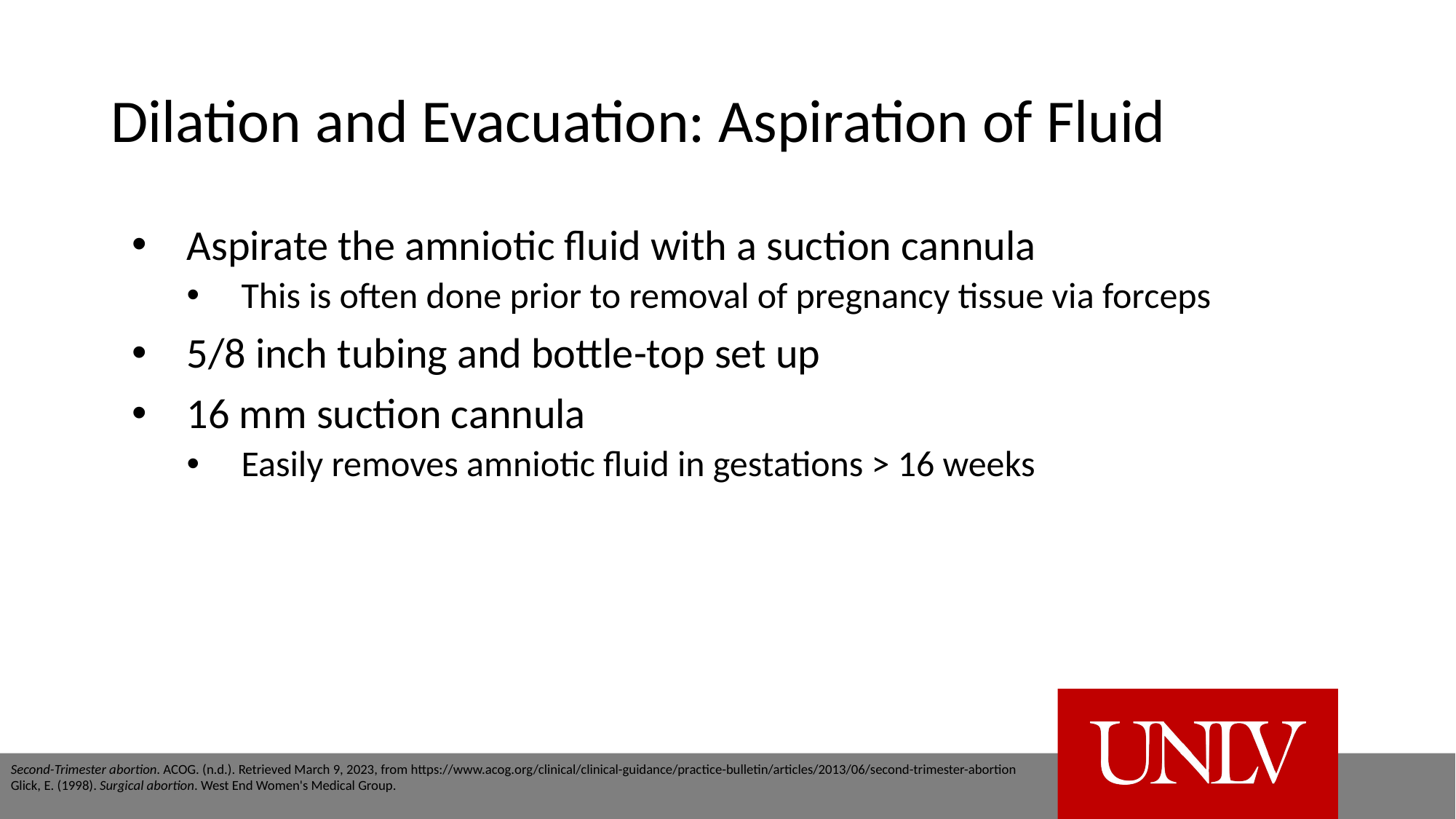

# Dilation and Evacuation: Aspiration of Fluid
Aspirate the amniotic fluid with a suction cannula
This is often done prior to removal of pregnancy tissue via forceps
5/8 inch tubing and bottle-top set up
16 mm suction cannula
Easily removes amniotic fluid in gestations > 16 weeks
Second-Trimester abortion. ACOG. (n.d.). Retrieved March 9, 2023, from https://www.acog.org/clinical/clinical-guidance/practice-bulletin/articles/2013/06/second-trimester-abortion
Glick, E. (1998). Surgical abortion. West End Women's Medical Group.

## Slide 26
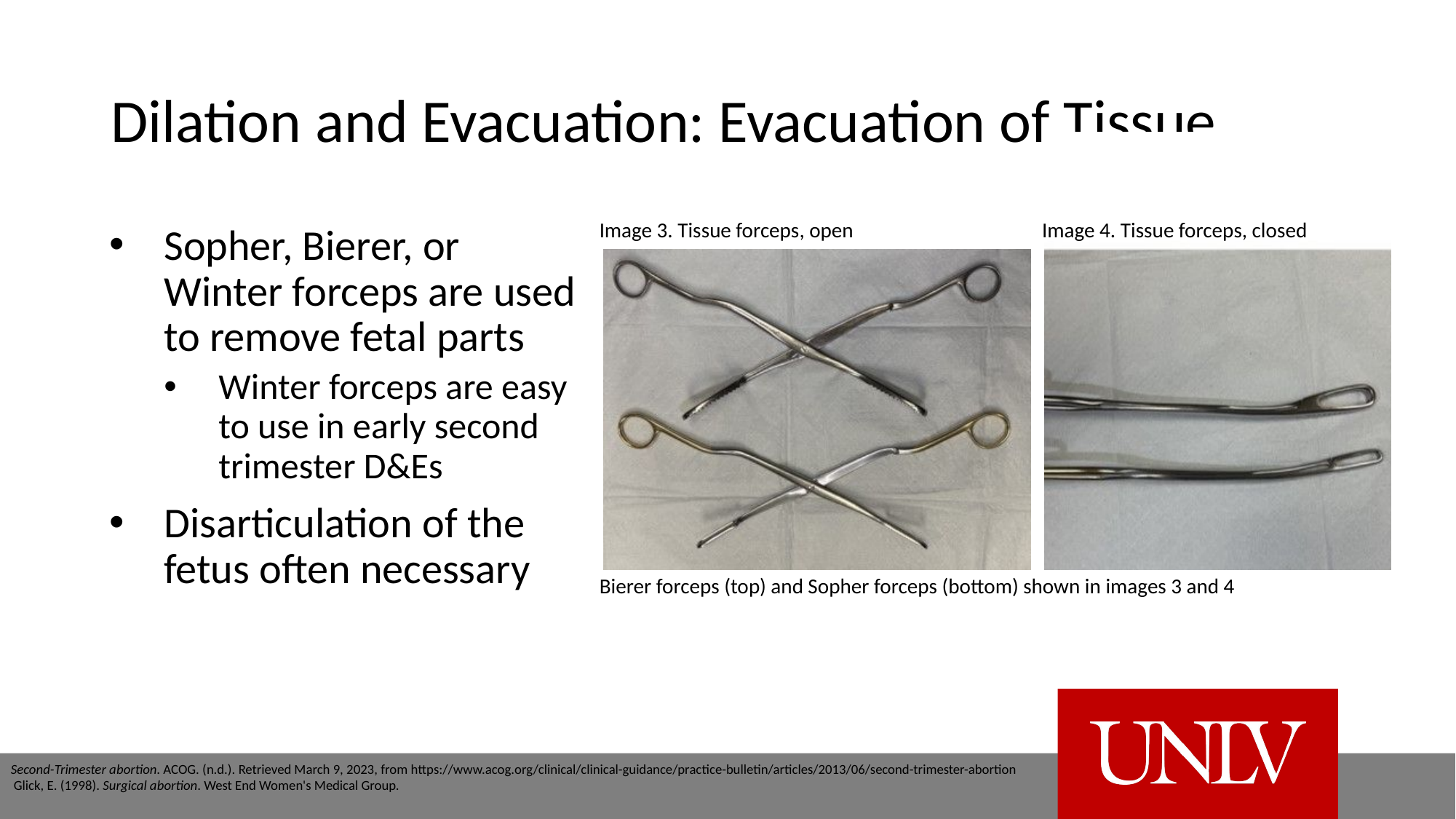

# Dilation and Evacuation: Evacuation of Tissue
Image 3. Tissue forceps, open Image 4. Tissue forceps, closed
Bierer forceps (top) and Sopher forceps (bottom) shown in images 3 and 4
Sopher, Bierer, or Winter forceps are used to remove fetal parts
Winter forceps are easy to use in early second trimester D&Es
Disarticulation of the fetus often necessary
Second-Trimester abortion. ACOG. (n.d.). Retrieved March 9, 2023, from https://www.acog.org/clinical/clinical-guidance/practice-bulletin/articles/2013/06/second-trimester-abortion
 Glick, E. (1998). Surgical abortion. West End Women's Medical Group.

## Slide 27
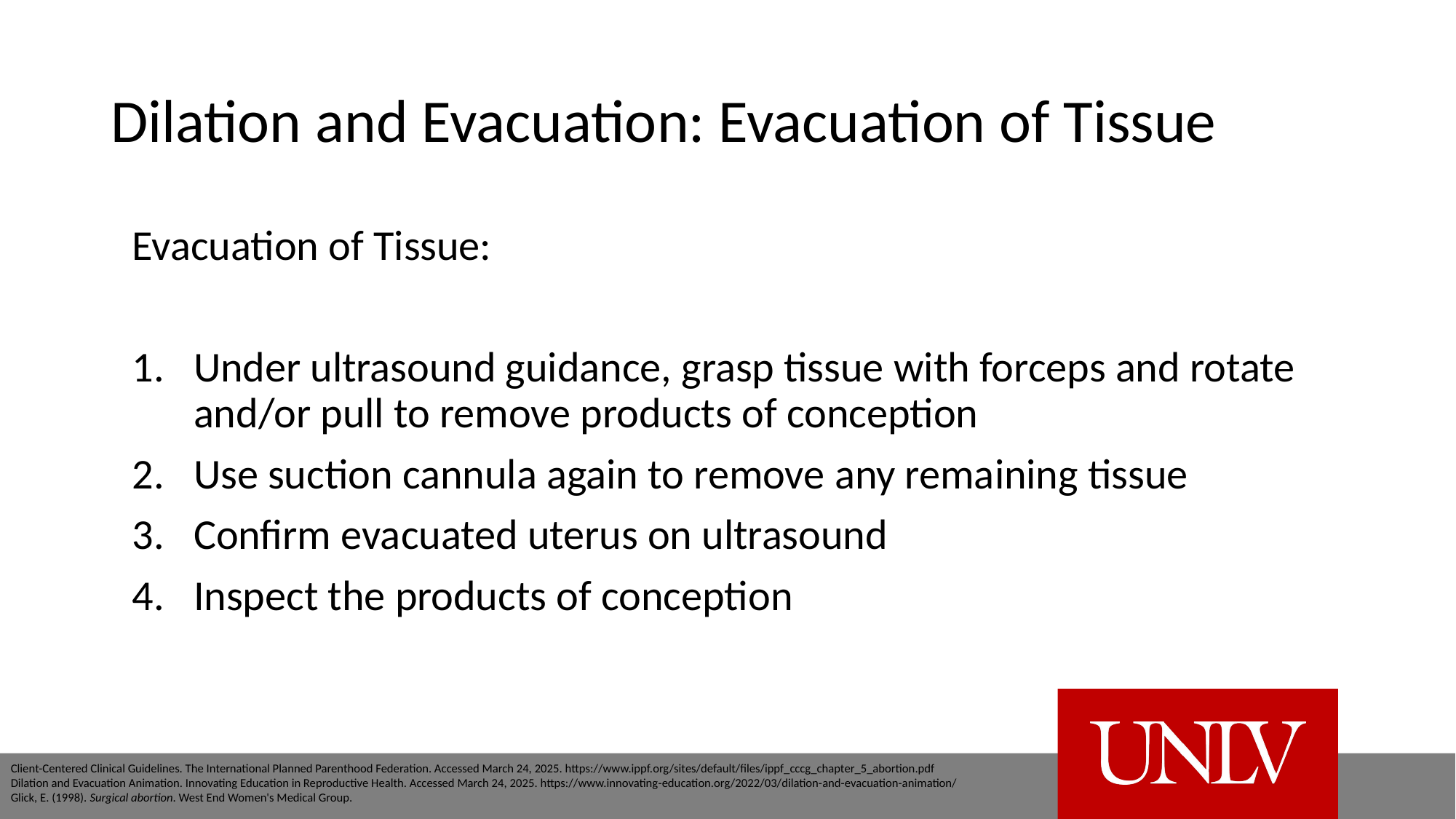

# Dilation and Evacuation: Evacuation of Tissue
Evacuation of Tissue:
Under ultrasound guidance, grasp tissue with forceps and rotate and/or pull to remove products of conception
Use suction cannula again to remove any remaining tissue
Confirm evacuated uterus on ultrasound
Inspect the products of conception
Client-Centered Clinical Guidelines. The International Planned Parenthood Federation. Accessed March 24, 2025. https://www.ippf.org/sites/default/files/ippf_cccg_chapter_5_abortion.pdf
Dilation and Evacuation Animation. Innovating Education in Reproductive Health. Accessed March 24, 2025. https://www.innovating-education.org/2022/03/dilation-and-evacuation-animation/
Glick, E. (1998). Surgical abortion. West End Women's Medical Group.

## Slide 28
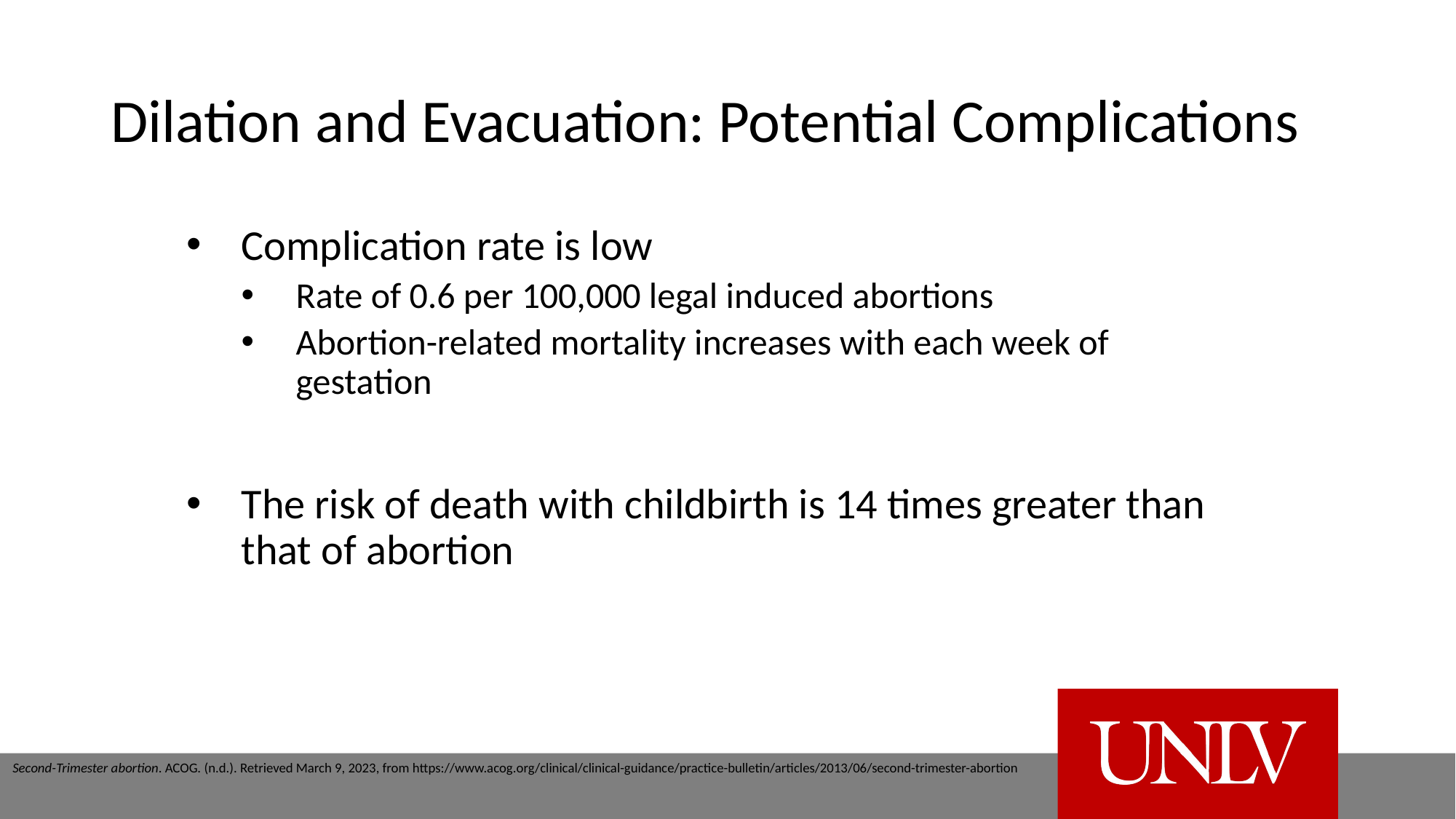

# Dilation and Evacuation: Potential Complications
Complication rate is low
Rate of 0.6 per 100,000 legal induced abortions
Abortion-related mortality increases with each week of gestation
The risk of death with childbirth is 14 times greater than that of abortion
Second-Trimester abortion. ACOG. (n.d.). Retrieved March 9, 2023, from https://www.acog.org/clinical/clinical-guidance/practice-bulletin/articles/2013/06/second-trimester-abortion

## Slide 29
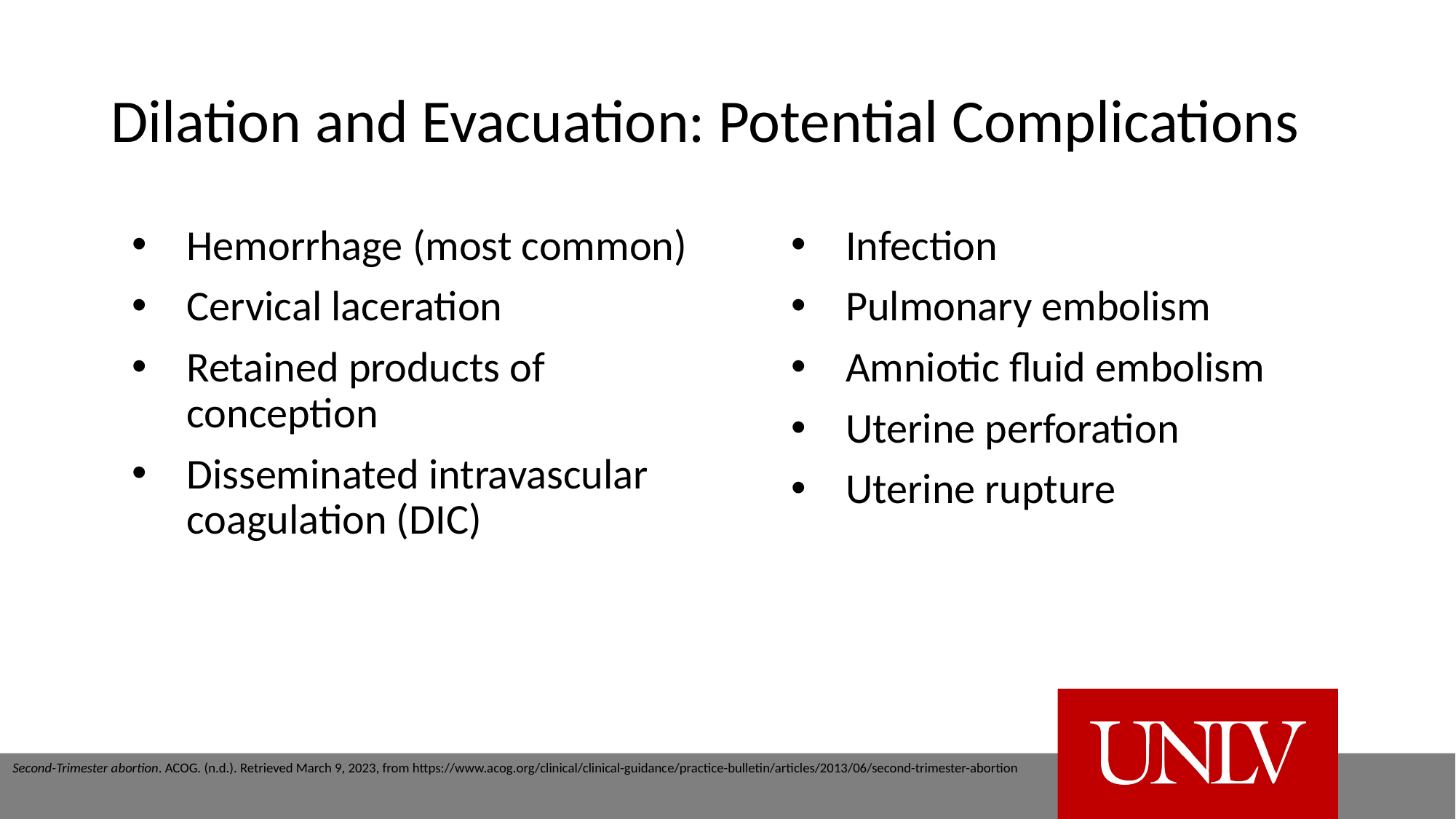

# Dilation and Evacuation: Potential Complications
Hemorrhage (most common)
Cervical laceration
Retained products of conception
Disseminated intravascular coagulation (DIC)
Infection
Pulmonary embolism
Amniotic fluid embolism
Uterine perforation
Uterine rupture
Second-Trimester abortion. ACOG. (n.d.). Retrieved March 9, 2023, from https://www.acog.org/clinical/clinical-guidance/practice-bulletin/articles/2013/06/second-trimester-abortion

## Slide 30
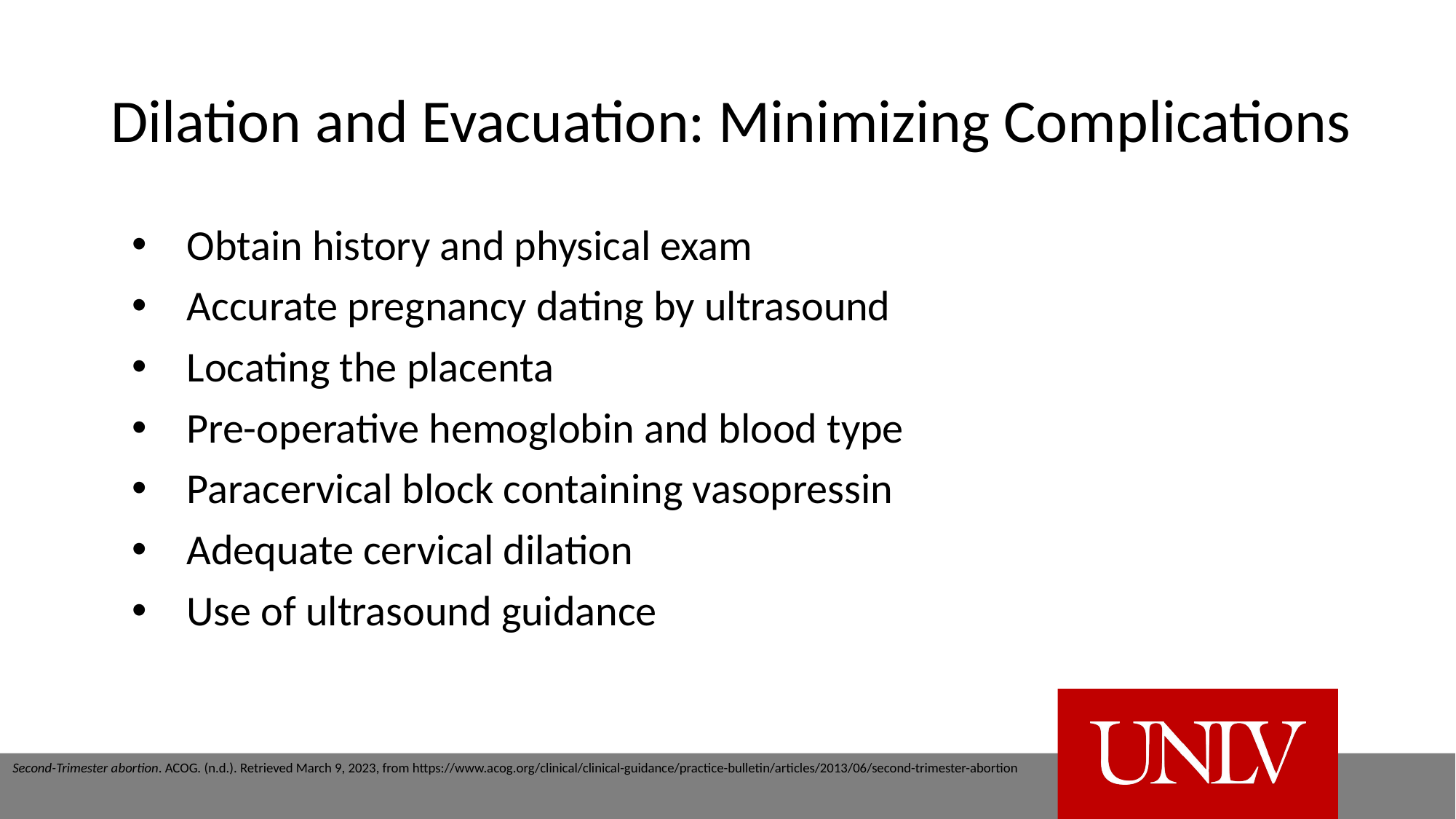

# Dilation and Evacuation: Minimizing Complications
Obtain history and physical exam
Accurate pregnancy dating by ultrasound
Locating the placenta
Pre-operative hemoglobin and blood type
Paracervical block containing vasopressin
Adequate cervical dilation
Use of ultrasound guidance
Second-Trimester abortion. ACOG. (n.d.). Retrieved March 9, 2023, from https://www.acog.org/clinical/clinical-guidance/practice-bulletin/articles/2013/06/second-trimester-abortion

## Slide 31
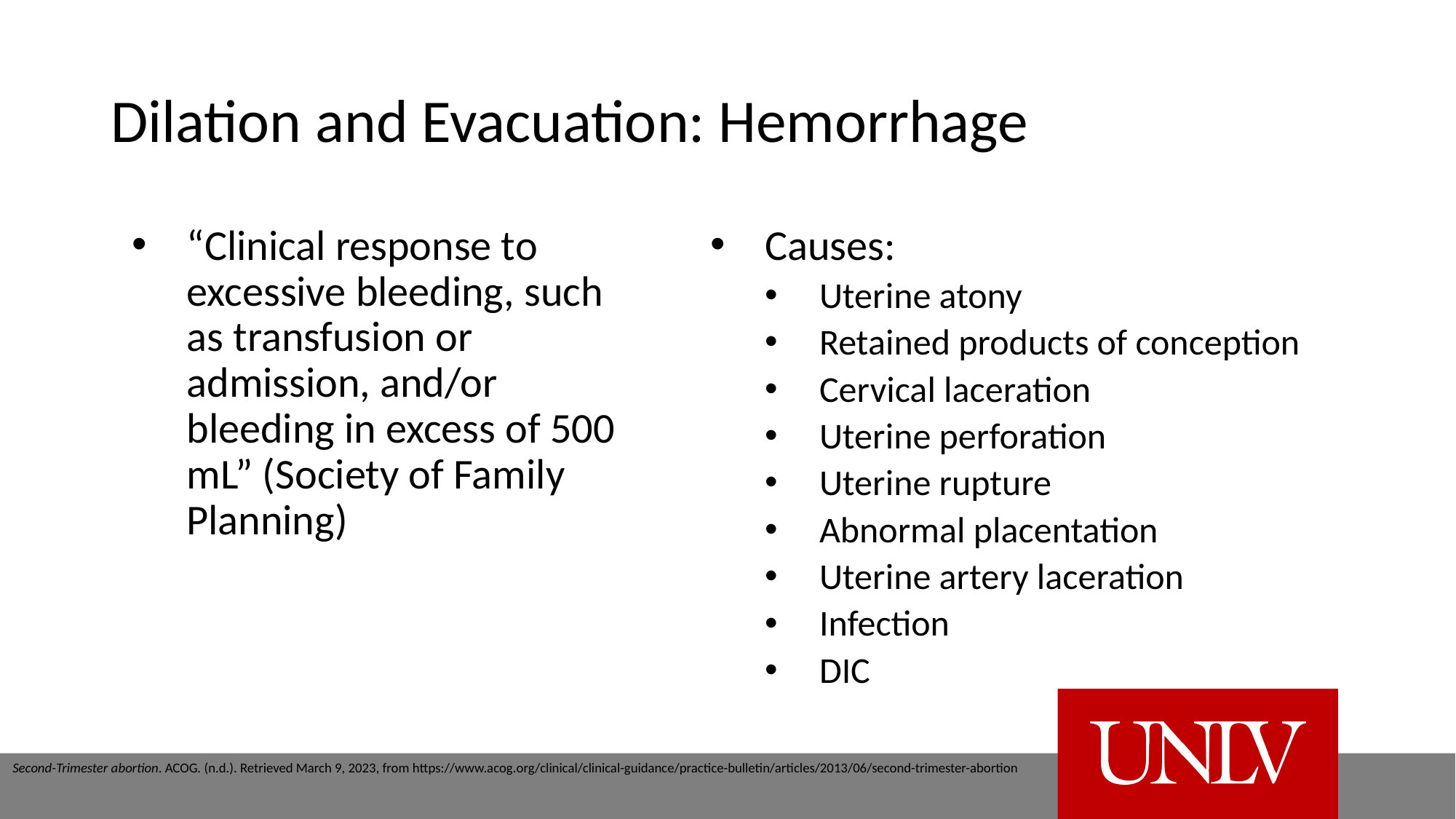

# Dilation and Evacuation: Hemorrhage
“Clinical response to excessive bleeding, such as transfusion or admission, and/or bleeding in excess of 500 mL” (Society of Family Planning)
Causes:
Uterine atony
Retained products of conception
Cervical laceration
Uterine perforation
Uterine rupture
Abnormal placentation
Uterine artery laceration
Infection
DIC
Second-Trimester abortion. ACOG. (n.d.). Retrieved March 9, 2023, from https://www.acog.org/clinical/clinical-guidance/practice-bulletin/articles/2013/06/second-trimester-abortion

## Slide 32
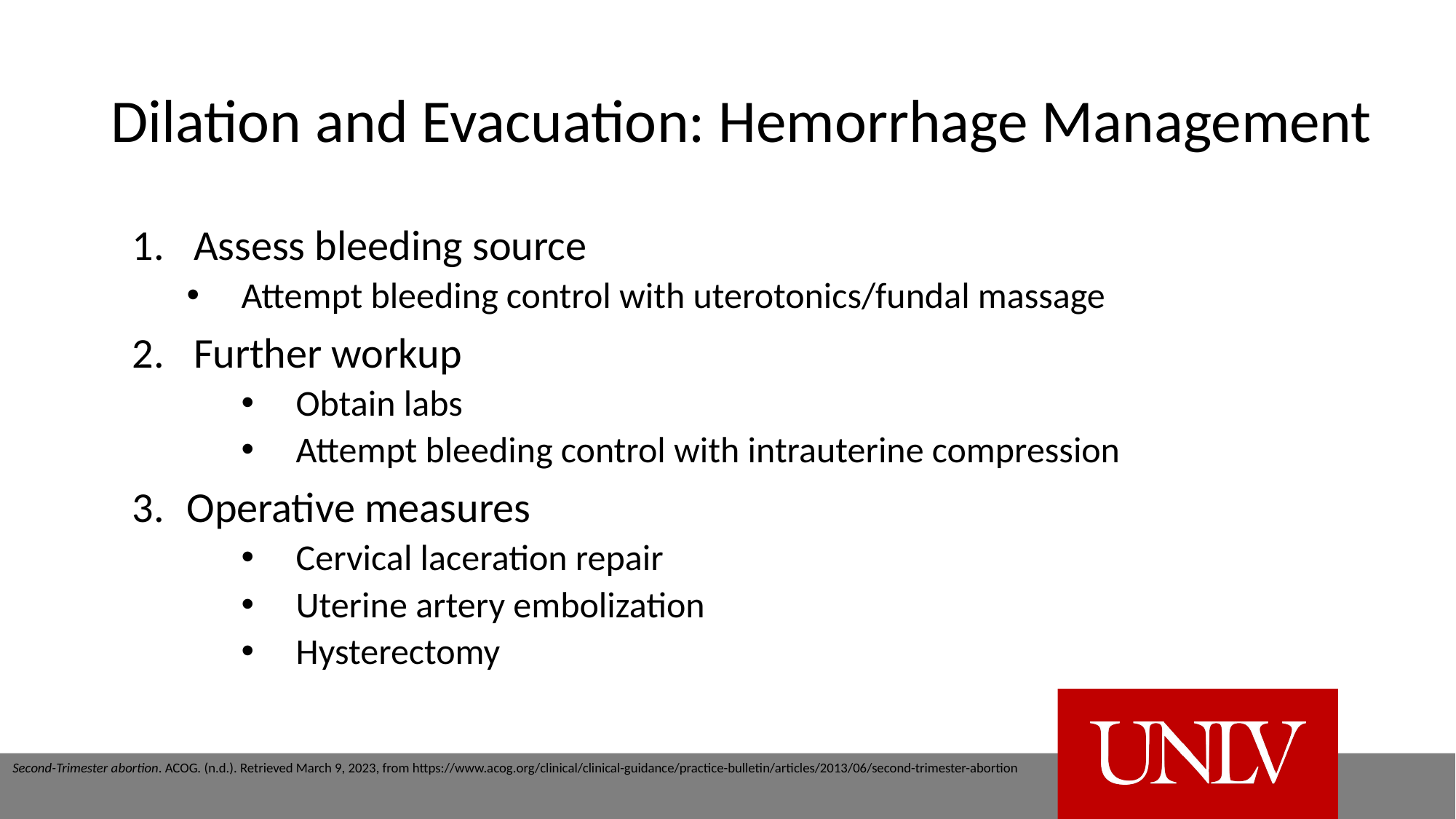

# Dilation and Evacuation: Hemorrhage Management
Assess bleeding source
Attempt bleeding control with uterotonics/fundal massage
Further workup
Obtain labs
Attempt bleeding control with intrauterine compression
Operative measures
Cervical laceration repair
Uterine artery embolization
Hysterectomy
Second-Trimester abortion. ACOG. (n.d.). Retrieved March 9, 2023, from https://www.acog.org/clinical/clinical-guidance/practice-bulletin/articles/2013/06/second-trimester-abortion

## Slide 33
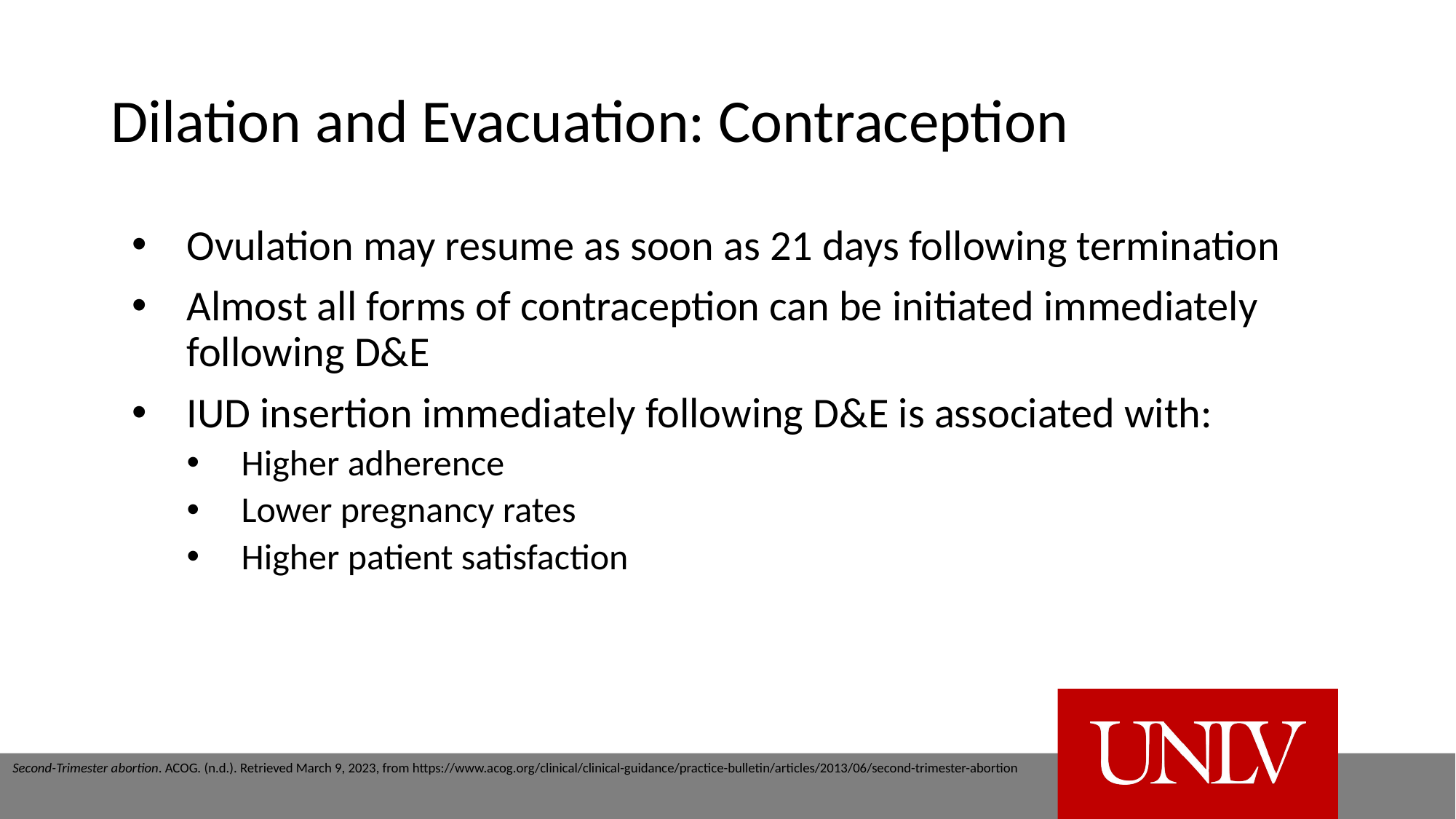

# Dilation and Evacuation: Contraception
Ovulation may resume as soon as 21 days following termination
Almost all forms of contraception can be initiated immediately following D&E
IUD insertion immediately following D&E is associated with:
Higher adherence
Lower pregnancy rates
Higher patient satisfaction
Second-Trimester abortion. ACOG. (n.d.). Retrieved March 9, 2023, from https://www.acog.org/clinical/clinical-guidance/practice-bulletin/articles/2013/06/second-trimester-abortion

## Slide 34
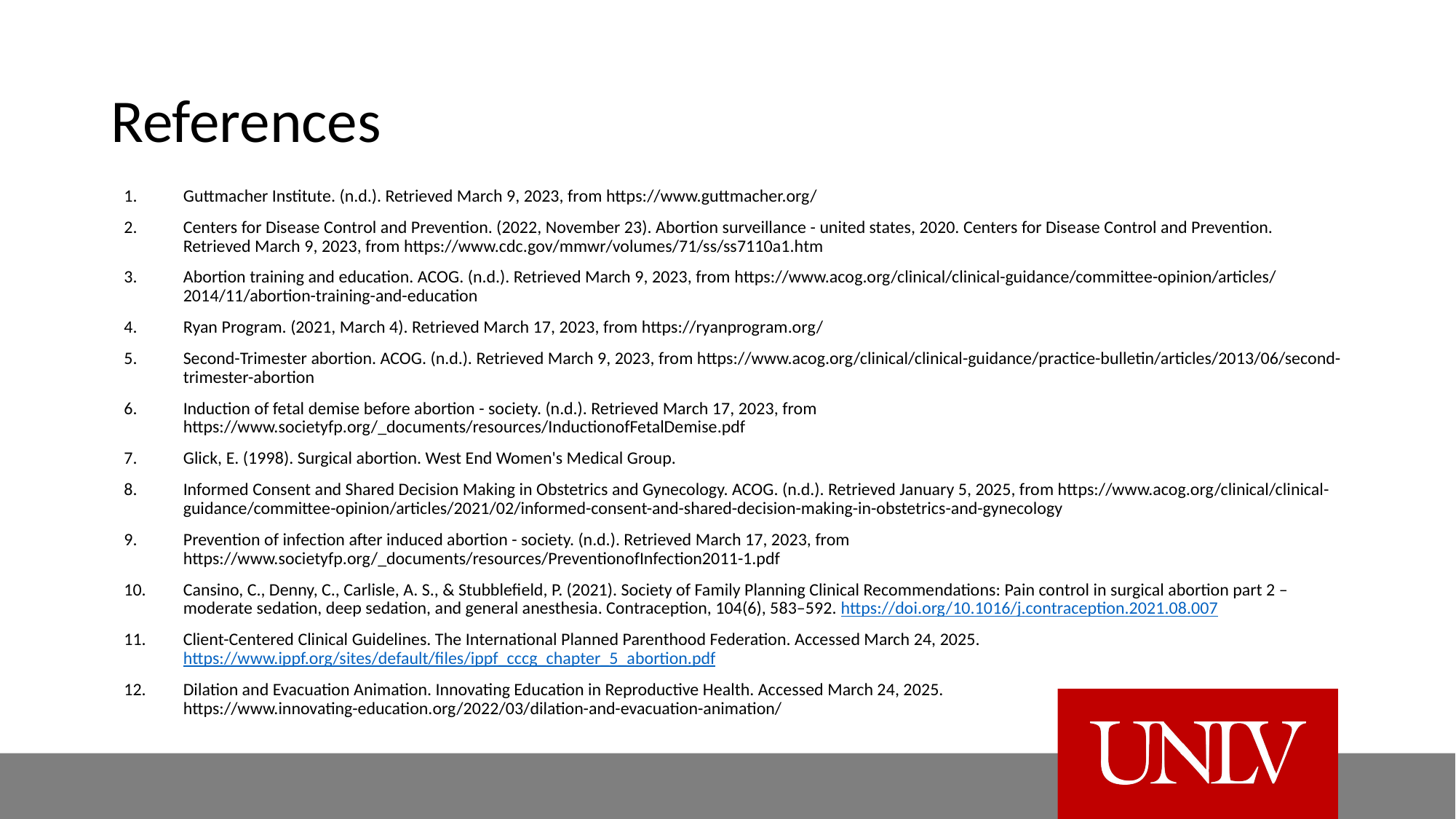

# References
Guttmacher Institute. (n.d.). Retrieved March 9, 2023, from https://www.guttmacher.org/
Centers for Disease Control and Prevention. (2022, November 23). Abortion surveillance - united states, 2020. Centers for Disease Control and Prevention. Retrieved March 9, 2023, from https://www.cdc.gov/mmwr/volumes/71/ss/ss7110a1.htm
Abortion training and education. ACOG. (n.d.). Retrieved March 9, 2023, from https://www.acog.org/clinical/clinical-guidance/committee-opinion/articles/2014/11/abortion-training-and-education
Ryan Program. (2021, March 4). Retrieved March 17, 2023, from https://ryanprogram.org/
Second-Trimester abortion. ACOG. (n.d.). Retrieved March 9, 2023, from https://www.acog.org/clinical/clinical-guidance/practice-bulletin/articles/2013/06/second-trimester-abortion
Induction of fetal demise before abortion - society. (n.d.). Retrieved March 17, 2023, from https://www.societyfp.org/_documents/resources/InductionofFetalDemise.pdf
Glick, E. (1998). Surgical abortion. West End Women's Medical Group.
Informed Consent and Shared Decision Making in Obstetrics and Gynecology. ACOG. (n.d.). Retrieved January 5, 2025, from https://www.acog.org/clinical/clinical-guidance/committee-opinion/articles/2021/02/informed-consent-and-shared-decision-making-in-obstetrics-and-gynecology
Prevention of infection after induced abortion - society. (n.d.). Retrieved March 17, 2023, from https://www.societyfp.org/_documents/resources/PreventionofInfection2011-1.pdf
Cansino, C., Denny, C., Carlisle, A. S., & Stubblefield, P. (2021). Society of Family Planning Clinical Recommendations: Pain control in surgical abortion part 2 – moderate sedation, deep sedation, and general anesthesia. Contraception, 104(6), 583–592. https://doi.org/10.1016/j.contraception.2021.08.007
Client-Centered Clinical Guidelines. The International Planned Parenthood Federation. Accessed March 24, 2025. https://www.ippf.org/sites/default/files/ippf_cccg_chapter_5_abortion.pdf
Dilation and Evacuation Animation. Innovating Education in Reproductive Health. Accessed March 24, 2025. https://www.innovating-education.org/2022/03/dilation-and-evacuation-animation/
