# Supplementary material for: Dilation and Evacuation Simulation Model for Learners and Providers Who Offer Abortion Care
Source: MedEdPORTAL. 2025 May 9;21:11525. doi: 10.15766/mep_2374-8265.11525 (PMC12062342; doi:10.15766/mep_2374-8265.11525)
Supplement: Supplementary file 1 — Simulation Materials and Assembly.docxPresimulation Survey.docxIntroductory Lecture.pptxD&E Simulation Demonstration Video.mp4Postsimulation Survey.docx [file mep_2374-8265.11525-s001.zip › _Educational Summary Report_11525.pdf]

MedEdPORTAL® | The AAMC Journal of Teaching and Learning Resources

Original Publication

Open Access

Dilation and Evacuation Simulation Model for Learners and Providers Who Offer Abortion Care

Haven Frazier, DO*, Leanne Free, MD, Shana Miles, MD, PhD, Matthew Vanbaaren, MD, Adam Levy, MD

*Corresponding author: frazierhaven@gmail.com

Abstract

Introduction: In the US, one in four women will have an abortion, and most OB/GYN physicians have had patients who required abortion care. Most second-trimester abortions in the US (95%) are performed via dilation and evacuation (D&E), which requires provider skill and competency. Barriers to obtaining abortion training include opt-in residency programs, location-based legal restrictions, and religiously affiliated institutions. Our D&E simulation is a cost-effective, realistic model. **Methods:** D&E models were assembled using juice containers, Cornish hens, and Sopher forceps. Thirty-five participants (medical students and OB/GYN residents) completed presimulation surveys and received a brief lecture about abortion demographics, techniques, and complications, followed by the hands-on simulation; 27 completed postsimulation surveys. Participants assessed their comfort levels in performing D&Es and recognizing postabortion complications, and their likelihood of performing D&Es in future clinical practice. **Results:** Comfort levels significantly improved pre- to postsimulation, increasing from 32% to 55% ($p < .001$) for participants reporting feeling *somewhat comfortable* or *extremely comfortable* performing D&Es, and increasing from 46% to 63% ($p < .01$) for participants reporting feeling *somewhat comfortable* or *extremely comfortable* recognizing postabortion complications after receiving the introductory lecture. Overall, participants indicated that the simulation was realistic (92%) and increased their knowledge (100%) and ability to perform D&Es (96%). **Discussion:** Our affordable and simple D&E model can be easily replicated and implemented for training in second-trimester D&E. This model can serve as a valuable and realistic tool for providers with restricted access to clinical abortion who need adjunct training, improving physician education and competency.

Keywords
Abortion, Dilation and Evacuation, OB/GYN, Simulation, Women's Health, Clinical/Procedural Skills Training, Gynecologic Surgery

Educational Objectives

By the end of this activity, learners will be able to:

1. Discuss abortion demographics in the US.
2. Discuss the types of abortion training in graduate and undergraduate medical education.
3. Discuss barriers to abortion training in the US.
4. Describe the pros and cons of medical versus surgical abortion in the second trimester.
5. Consent a patient for dilation and evacuation (D&E).
6. List the indications for D&E.
7. Describe how to prepare for and perform D&E.
8. Practice a key component of D&E manual skills (fetal evacuation) on simulation models.
9. Describe and demonstrate methods of handling forceps during D&E.
10. List postabortion complications.
11. Define postabortion hemorrhage and discuss the management of this complication.
12. Discuss methods of post-D&E contraception.

Citation:
Frazier H, Free L, Miles S, Vanbaaren M, Levy A. Dilation and evacuation simulation model for learners and providers who offer abortion care. *MedEdPORTAL*. 2025;21:11525.
https://doi.org/10.15766/mep_2374-8265.11525

Introduction

Abortion care is an essential and common part of reproductive health care. In the US, one in four women have an abortion in their lifetimes, and almost all OB/GYN physicians have had patients who required some sort of uterine evacuation.[1,2] Although 90% of abortions occur in the first trimester, 95% of those that occur at later gestational ages in the US are performed via D&E, which requires more provider skill and competency to perform.[3,4] When compared to medical management, surgical management is often faster, associated with fewer complications,

Copyright © 2025 Frazier et al. This is an open-access publication distributed under the terms of the Creative Commons Attribution license.

more cost-effective, and more often preferred by patients undergoing second-trimester abortion.[4]

Providers often face barriers when it comes to obtaining abortion training, including opt-in residency programs, legal restrictions based on location of practice, and institutions with religious affiliations.[5] In order to become certified with the American Board of Obstetrics and Gynecology, OB/GYNs must complete, at a minimum, the equivalent of two 4-week blocks of family planning during residency, which should include structured curriculum and clinical experiences in contraception, surgical sterilization, patient counseling about medical and surgical abortions, and management of abortion complications. Augmenting clinical experiences with simulation is encouraged.[6] Despite this, only four in 10 US OB/GYN physicians reported providing routine abortion care for their patients, and only 64% of OB/GYN residency program directors reported providing routine abortion training for their residents.[2] The American College of Obstetricians and Gynecologists supports increasing the pool of abortion providers to include family medicine physicians and advanced practice clinicians, as well as integrating routine abortion training into resident and medical student education.[5]

Most existing abortion simulation models are designed for first-trimester abortions; fewer exist for developing skills in D&E in the second trimester. To our knowledge, one other D&E simulation model has been published in *MedEdPORTAL*;[7] this model requires D&E simulation using a high-fidelity birthing mannequin, among other pieces of specialty medical equipment. While this design simulates clinical scenarios and postabortion complications, it may also be impractical or inaccessible for providers outside of an academic setting. A lower-fidelity D&E simulation model similar to ours was published in 2022;[8] however, this model uses less realistic materials for the products of conception. Additionally, the model was part of a multiphase study introducing D&E to the community, and descriptions of the simulation model itself are limited.[8] Another D&E simulation model employs large plastic bottles for the uterus, but the fetal parts and endometrium were found to be less realistic by study participants.[9]

Our simulation was originally designed in 2020 and the responses were universally positive among the University of Nevada, Las Vegas (UNLV) and Mountain View Hospital OB/GYN residents. It employs a low-budget (approximately $8 per model and $92 for bulk supplies), easy to assemble (approximately 30 minutes), and realistic design developed for learners and providers to practice D&Es, despite any legal or institutional barriers they may face. These features accommodate institutions with limited resources, time, or budgets, while also providing realistic tactile sensation to simulate D&E procedures.

Methods

The OB/GYN residency program at UNLV is a combined civilian and military program with a total of 16 civilian residents and eight military residents. Clinical education primarily takes place at a private hospital and the university-associated hospital and outpatient clinics, with occasional clinical duties performed at Nellis Air Force Base. The UNLV OB/GYN residency program is a participating institution in the Kenneth J. Ryan Training Program in Abortion and Family Planning, which was designed to help residency programs integrate family planning (including abortion care) into resident education.[2] Family planning rotations at UNLV are longitudinal across all 4 years, and abortion care training is opt-out. Residents spend approximately 2 weeks at the family planning clinic during each year of residency, where they take part in providing medication abortion, procedural abortion, contraception, and management of abortion-related complications. During this study period, most residents participated in full-spectrum abortion care and no residents opted out. They had opportunities to start performing parts of D&Es during their first year of residency, and later performed D&Es fully during the remainder of their residency training. Third- and fourth-year medical students can elect to rotate at the family planning clinic, where they also learn full-spectrum family planning care.

This study received an institutional review board exempt status from the UNLV Office of Research Integrity–Human Subjects. Our D&E simulation was held during a single 1-hour didactics session for the OB/GYN residents and the third- and fourth-year medical students who were completing their OB/GYN clerkship at that time. The D&E models were assembled ahead of time (Appendix A) and Sopher forceps were borrowed from the family planning clinic and the university hospital operating room. Each D&E model was set up on an absorbent pad on a flat, narrow table. Gloves and Sopher forceps were available at each of these stations.

The following materials were required to create the D&E simulation models (assembled ahead of time; Appendix A) and to complete the activity:

- Plastic juice carton (1 gallon), with neck opening measuring approximately 1.5 inches (3.8 centimeters) in diameter
- Utility knife
- Tape measure
- Carpet foam (or similar spongy material)
- Scissors
- Double-sided tape

Copyright © 2025 Frazier et al. This is an open-access publication distributed under the terms of the Creative Commons Attribution license.

- Duct tape
- Cornish hen (raw)
- Sopher forceps
- Gloves (recommended)
- Absorbent pad or plastic sheet (recommended)
- Flat surface

To start the activity, residents and medical students completed a presimulation survey (Appendix B) on the Qualtrics platform. The survey assessed their preexisting knowledge surrounding second-trimester abortion and also asked participants to indicate their comfort level with performing D&Es and being able to recognize postabortion complications, with response options of *extremely uncomfortable*, *somewhat uncomfortable*, *neither comfortable nor uncomfortable*, *somewhat comfortable*, and *extremely comfortable*.

Next, participants received a brief introductory lecture (Appendix C), which outlined abortion demographics in the US, second-trimester abortion techniques including D&Es, and complications of procedural abortions. This was followed by dividing the participants into pairs at each D&E station. One of the participants practiced the D&E technique while the other held and secured the simulation model in place from the opposite side of the table. Throughout the simulation activity, family planning faculty were present to instruct on technique, although instruction can also be received from OB/GYN generalists or maternal-fetal medicine specialists who perform D&Es as a part of their clinical practice.

The following methods for practicing D&E technique (Appendix D) were recommended:

- The person performing the D&E should stand in front of the neck of the carton and use grasping forceps to methodically remove the hen (and loose foam) in pieces.
- Another person should stand at the base of the carton to firmly hold it in place against the table.
- Introduce the forceps into the carton.
    - Forceps are generally introduced and opened in an anterior-posterior direction, so both jaws can be visualized on transabdominal ultrasound. They may also be opened horizontally; however, both jaws cannot be visualized simultaneously on ultrasound in this orientation.[10,11]
    - Drop your hands if needed to match the angle of the uterus, if positioned anteriorly.[10]
    - Grasp tissue and rotate prior to pulling to reduce complications involving the uterine wall.
    - [Optional] Hanson's Maneuver: When ultrasound is not available, place one hand on the uterine fundus (base of the carton) and do not allow the forceps to pass your hand (this has limited utility in obese patients).[12]
- Once all of the hen (and loose foam) has been removed, the inside of the carton should be examined to evaluate for any pieces of adhered carpet foam that have been removed. This would simulate injured uterine tissue.

At the conclusion of the activity, the residents and medical students completed a postsimulation survey (Appendix E). The total duration of the lecture and simulation was approximately 1.5 hours. Results were analyzed using descriptive statistics and chi-squared tests.

Results

In total, 35 people completed the presimulation survey and participated in the simulation activity, and 27 completed the postsimulation survey. Simulation participants included third-year medical students, fourth-year medical students, and residents at all four training levels (Table 1). Of all simulation participants, 66% reported having participated in a D&E simulation in the past, while 34% had not. Similarly, 66% of participants reported having performed D&Es in an outpatient or inpatient clinical setting in the past, while 34% had not.

Participants' comfort level in their ability to perform D&Es increased significantly pre- to postsimulation, with participants indicating a comfort level of *somewhat comfortable* or *extremely comfortable* increasing from 32% to 55% ($p < .001$). Moreover, participants reported feeling more comfortable recognizing postabortion complications after receiving the introductory lecture, increasing from 46% to 63% of participants ($p < .01$; Table 2).

Table 1. Demographics and Number of Participants ($N = 35$)

	n						
**Survey**	**MS 3**	**MS 4**	**PGY 1**	**PGY 2**	**PGY 3**	**PGY 4**	**Total**
Presimulation	12	4	6	5	4	4	35
Postsimulation	7	4	6	2	4	4	27

Abbreviation: MS, medical student.

Copyright © 2025 Frazier et al. This is an open-access publication distributed under the terms of the Creative Commons Attribution license.

Table 2. Participant Comfort Level in Skills

Skill	*n* (%)					*p*
	Extremely Comfortable	Somewhat Comfortable	Neither	Somewhat Uncomfortable	Extremely Uncomfortable	
Performing D&E						
Presimulation[a]	2 (6)	9 (26)	2 (6)	2 (6)	20 (57)	<.001
Postsimulation[b]	2 (7)	13 (48)	7 (26)	4 (15)	1 (4)	<.001
Recognizing complications						
Presimulation[a]	1 (3)	15 (43)	3 (9)	6 (17)	10 (29)	<.01
Postsimulation[b]	3 (11)	14 (52)	8 (30)	2 (7)	0 (0)	<.01

Abbreviation: D&E, dilation and evacuation.
[a]$N = 35$.
[b]$N = 27$.

Participants were also asked if they were more likely to perform D&Es in future practice if they were to receive more simulated training during residency. Students who did not intend to pursue a specialty in OB/GYN chose *not applicable*. The percentage of participants who responded *yes* to indicate they were likely to perform D&Es in future if given more simulated training increased from 60% to 67% pre- to postsimulation, and the percentage who responded *no* or *unsure* decreased from 40% to 33% pre- to postsimulation ($p = .62$). When asked if they were more likely to perform D&Es in future practice if they were to receive more clinical training (as opposed to simulated training) during residency, the percentage of participants who responded *yes* increased from 62% to 67%, and the percentage who responded *no* or *unsure* decreased from 38% to 33% ($p = .94$). These response changes from pre- to postsimulation were not statistically significant (Table 3).

After completion of the simulation, the participants were asked their opinions on the simulation experience. Overall, most participants felt that the simulation was realistic (92%) and increased their knowledge (100%) and their ability to perform D&Es (96%; Table 3).

Table 3. Participant Pre- and Postsimulation Feedback

Survey Question	*n* (%)		*p*
	Pre[a]	Post[b]	
Would perform future D&Es after more simulation training			
Yes	15 (60)	14 (67)	.62
No or Unsure	10 (40)	7 (33)	.62
Would perform future D&Es after more clinical training			
Yes	16 (62)	14 (67)	.94
No or Unsure	10 (38)	7 (33)	.94
Simulation was realistic			
Yes		25 (92)	
No or Unsure		2 (7)	
Simulation increased participant knowledge			
Yes		27 (100)	
No or Unsure		0 (0)	
Simulation increased participant ability			
Yes		26 (96)	
No or Unsure		1 (4)	

Abbreviation: D&E, dilation and evacuation.
[a]$N = 35$.
[b]$N = 27$.

Discussion

It is imperative for OB/GYN physicians to become competent in D&E. Unfortunately, with increased legal restrictions and institutional barriers based on location of practice, there is increasingly limited opportunity to use and practice this skill set until it becomes medically indicated, at which point the clinical scenario may be urgent or emergent. The simulation presented is a low-budget and realistic model that can be easily implemented for supplemental training in D&E.

Our D&E model can be easily replicated, unlike other models which are cost-prohibitive or require advanced equipment. The model is easily assembled with few supplies, some of which are a one-time purchase (utility knife, scissors, juice containers), while others can be purchased in bulk for multiple uses (carpet foam, gloves, absorbent pads). Cornish hens (or similar poultry products) are available at most grocery stores. Obtaining Sopher forceps proved to be somewhat more challenging; however, these can be either obtained through a one-time purchase for multiple uses or, depending on local resources, borrowed from nearby facilities.

Existing D&E simulations, endorsed by American College of Obstetricians and Gynecologists and Innovating Education in Reproductive Health, range from low-fidelity models such as our own to high-fidelity models that simulate emergency scenarios. These low-fidelity pelvic models are made from melons, glass jars, plastic bottles, or neoprene, and the fetal models include a variety of objects including dried pasta, pepperoni sticks, mushrooms, eggs, ping-pong balls, cat toys, modeling clay, washcloths, facial tissue, and velcro.[13-16] While study participants have found plastic bottles to be more realistic compared to other models, there have been varied responses

Copyright © 2025 Frazier et al. This is an open-access publication distributed under the terms of the Creative Commons Attribution license.

to the tactile sensation of removing these aforementioned products and how well they simulate removing fetal parts.[9] The use of Cornish hens in our model addresses these concerns by providing a more realistic tactile sensation and requires participants to disarticulate the hen prior to removing it from the container.

Overall, the simulation was successful in increasing learner knowledge and confidence in performing D&Es. Limitations to this simulation include a lack of full postsimulation survey participation (27 of 35 participants completed the second survey), and the large proportion of participants who were medical students who may not practice in a specialty that provides abortion care.

While simulation training does not entirely replace clinical experience, this model could serve as a valuable adjunct tool for physicians and other providers with restricted access to clinical abortion training. This is especially important for residency programs that only offer opt-in training or programs that do not offer any abortion training, as these physicians are still likely to encounter indicated terminations even if they reside in restrictive states. Incorporating D&E simulation into OB/GYN residency training could standardize and improve physician education and competency.

Appendices

A. Simulation Materials and Assembly.docx
B. Presimulation Survey.docx
C. Introductory Lecture.pptx
D. D&E Simulation Demonstration Video.mp4
E. Postsimulation Survey.docx

All appendices are peer reviewed as integral parts of the Original Publication.

Haven Frazier, DO: Fourth-Year Resident, Department of Gynecologic Surgery and Obstetrics, Kirk Kerkorian School of Medicine at UNLV and Nellis Air Force Base

Leanne Free, MD: Assistant Professor, Department of Gynecologic Surgery and Obstetrics, Kirk Kerkorian School of Medicine at UNLV

Shana Miles, MD, PhD: Associate Professor, Department of Gynecologic Surgery and Obstetrics, Uniformed Services University of the Health Sciences F. Edward Hebert School of Medicine and Nellis Air Force Base

Matthew Vanbaaren, MD: First-Year Resident, Department of Gynecologic Surgery and Obstetrics, Kirk Kerkorian School of Medicine at UNLV

Adam Levy, MD: Associate Professor, Department of Gynecologic Surgery and Obstetrics, Kirk Kerkorian School of Medicine at UNLV

Disclosures
None to report.

Funding/Support
None to report.

Prior Presentations
Williams J, Simmons A, Free L, Levy AV. Dilation and evacuation (D&E) simulation. Poster presented at: 2022 American College of Obstetricians and Gynecologists Armed Forces District Annual District Meeting; October 18, 2022; Henderson, NV.

Ethical Approval
The Kirk Kerkorian School of Medicine at University of Nevada, Las Vegas Office of Research Integrity–Human Subjects reviewed this project.

References

1. *Induced Abortion in the United States*. Guttmacher Institute; 2019. Accessed April 8, 2025. https://www.guttmacher.org/pubs/fb_induced_abortion.pdf
2. About the Ryan Program. Ryan Program. Accessed April 8, 2025. https://ryanprogram.org/home/overview/
3. Kortsmit K, Nguyen AT, Mandel MG, et al. Abortion Surveillance—United States, 2020. MMWR Surveillance Summaries. 2022;71(10):1-27. https://doi.org/10.15585/mmwr.ss7110a1
4. Practice bulletin no. 135: second-trimester abortion. *Obstet Gynecol*. 2013;121(6):1394-1406. https://doi.org/10.1097/01.AOG.0000431056.79334.cc
5. Committee opinion no. 612: abortion training and education. *Obstet Gynecol*. 2014;124(5):1055-1059. https://doi.org/10.1097/01.AOG.0000456327.96480.18
6. OB GYN standards for certification clarification. American Board of Obstetrics and Gynecology. Accessed April 8, 2025. https://www.abog.org/specialty-certification/ob-gyn-standards-for-certification-clarification
7. Schwartz LN, Pelletier A, Goldberg AB, et al. Second-trimester dilation and evacuation: a simulation-based team training curriculum. *MedEdPORTAL*. 2023;19:11336. https://doi.org/10.15766/mep_2374-8265.11336
8. Stifani BM, Mei Hwang S, Rodrigues Catani R, Borges Martins da Silva Paro H, Benfield N. Introducing the dilation and evacuation technique in Brazil: lessons learned from an international partnership to expand options for Brazilian women and girls. *Front Glob Womens Health*. 2022;3:811412. https://doi.org/10.3389/fgwh.2022.811412

Copyright © 2025 Frazier et al. This is an open-access publication distributed under the terms of the Creative Commons Attribution license.

9. Baldwin MK, Chor J, Chen BA, Edelman AB, Russo J. Comparison of 3 dilation and evacuation technical skills models. *J Grad Med Educ*. 2013;5(4):662-664. https://doi.org/10.4300/JGME-D-13-00049.1

10. *Client-Centered Clinical Guidelines for Sexual and Reproductive Healthcare*. International Planned Parenthood Federation; 2022. Accessed March 24, 2025. https://www.ippf.org/sites/default/files/ippf_client-centred_clinical_guidelines_complete.pdf

11. Lohr PA. Dilation and evacuation animation. Innovating Education in Reproductive Health. Accessed April 8, 2025. https://www.innovating-education.org/2022/03/dilation-and-evacuation-animation/

12. Glick E. *Surgical Abortion*. West End Women's Medical Group; 1998.

13. Curriculum: Simulations Working Group—second-trimester dilation and evacuation. American College of Obstetrics and Gynecologists. Accessed March 24, 2025. https://www.acog.org/education-and-events/simulations/scog019/simulation

14. Costescu D. Multiple-module simulation of dilation and evacuation facilitation guide. Innovating Education in Reproductive Health. Accessed April 8, 2025. https://www.innovating-education.org/2016/03/multiple-module-simulation-of-dilation-and-evacuation-facilitation-guide/

15. Payne C. Low-tech D&E training model instructional video. Innovating Education in Reproductive Health. Accessed April 8, 2025. https://www.innovating-education.org/2016/03/low-tech-de-training-model-instructional-video/

16. Bartz D. D&E capstone experience coupled with emergency scenario. Innovating Education in Reproductive Health. Accessed March 24, 2025. https://www.innovating-education.org/2016/03/de-capstone-experience-coupled-with-emergency-scenario/

Received: July 7, 2024
Accepted: March 31, 2025
Published: May 9, 2025

Copyright © 2025 Frazier et al. This is an open-access publication distributed under the terms of the Creative Commons Attribution license.
